# CircDOCK7 facilitates the proliferation and adipogenic differentiation of chicken abdominal preadipocytes through the gga-miR-301b-3p/*ACSL1* axis

**DOI:** 10.1186/s40104-023-00891-8

**Published:** 2023-07-06

**Authors:** Weihua Tian, Ye Liu, Wenhui Zhang, Ruixue Nie, Yao Ling, Bo Zhang, Hao Zhang, Changxin Wu

**Affiliations:** National Engineering Laboratory for Animal Breeding, Beijing Key Laboratory for Animal Genetic Improvement, College of Animal Science and Technology, China Agricultural University, Beijing, 100193 China

**Keywords:** Abdominal fat deposition, Adipogenesis, Chickens, CircDOCK7, Competing endogenous RNA, MiRNA sponge

## Abstract

**Background:**

Abdominal fat deposition depends on both the proliferation of preadipocytes and their maturation into adipocytes, which is a well-orchestrated multistep process involving many regulatory molecules. Circular RNAs (circRNAs) have emergingly been implicated in mammalian adipogenesis. However, circRNA-mediated regulation in chicken adipogenesis remains unclear. Our previous circRNA sequencing data identified a differentially expressed novel circRNA, 8:27,886,180|27,889,657, during the adipogenic differentiation of chicken abdominal preadipocytes. This study aimed to investigate the regulatory role of circDOCK7 in the proliferation and adipogenic differentiation of chicken abdominal preadipocytes, and explore its molecular mechanisms of competing endogenous RNA underlying chicken adipogenesis.

**Results:**

Our results showed that 8:27,886,180|27,889,657 is an exonic circRNA derived from the head-to-tail splicing of exons 19–22 of the dedicator of cytokinesis 7 (*DOCK7*) gene, abbreviated as circDOCK7. CircDOCK7 is mainly distributed in the cytoplasm of chicken abdominal preadipocytes and is stable because of its RNase R resistance and longer half-life. CircDOCK7 is significantly upregulated in the abdominal fat tissues of fat chickens compared to lean chickens, and its expression gradually increases during the proliferation and adipogenic differentiation of chicken abdominal preadipocytes. Functionally, the gain- and loss-of-function experiments showed that circDOCK7 promoted proliferation, G0/G1- to S-phase progression, and glucose uptake capacity of chicken abdominal preadipocytes, in parallel with adipogenic differentiation characterized by remarkably increased intracellular lipid droplet accumulation and triglyceride and acetyl coenzyme A content in differentiated chicken abdominal preadipocytes. Mechanistically, a pull-down assay and a dual-luciferase reporter assay confirmed that circDOCK7 interacted with gga-miR-301b-3p, which was identified as an inhibitor of chicken abdominal adipogenesis. Moreover, the *ACSL1* gene was demonstrated to be a direct target of gga-miR-301b-3p. Chicken ACSL1 protein is localized in the endoplasmic reticulum and mitochondria of chicken abdominal preadipocytes and acts as an adipogenesis accelerator. Rescue experiments showed that circDOCK7 could counteract the inhibitory effects of gga-miR-301b-3p on *ACSL1* mRNA abundance as well as the proliferation and adipogenic differentiation of chicken abdominal preadipocytes.

**Conclusions:**

CircDOCK7 serves as a miRNA sponge that directly sequesters gga-miR-301b-3p away from the *ACSL1* gene, thus augmenting adipogenesis in chickens. These findings may elucidate a new regulatory mechanism underlying abdominal fat deposition in chickens.

**Supplementary Information:**

The online version contains supplementary material available at 10.1186/s40104-023-00891-8.

## Background

Chickens are the most economically desirable agricultural poultry worldwide because of their meat and egg production. Intensive genetic selection for body weight and growth rate in broilers and laying chickens has tremendously improved their growth performance and reduced market age but is accompanied by excessive body fat disposition, especially abdominal fat. Excessive abdominal fat deposition is one of the main problems in the poultry industry, as it not only reduces feed efficiency, carcass yield, reproductive performance, and consumer preference but also increases the difficulty in meat processing as well as nitrogen and phosphate content in the excrement, thus resulting in considerable obstacles to profitable farming and environmental pollution [1–3]. Abdominal fat weight and percentage are direct indexes of abdominal fat deposition in chickens but are not suitable for indirect in vivo measurement. Chicken abdominal fat weight and percentage have high heritability and strong positive genetic correlation, indicating that the genetic approach is the most effective way to reduce excessive abdominal fat deposition in chickens [3, 4]. Therefore, it is of great significance to determine the crucial mediators of abdominal fat deposition, and thus contribute to the genetic improvement of excessive abdominal fat in chickens and breeding of lean-line broilers.

Abdominal fat deposition depends on the proliferation (number) of preadipocytes and their maturation (size) into adipocytes, which is a precisely orchestrated process regulated by many regulatory molecules, including protein-coding genes, transcription factors, epigenetic modifiers, and non-coding RNAs (ncRNAs). Several classes of ncRNAs, such as long non-coding RNAs (lncRNAs), circular RNAs (circRNAs), microRNAs (miRNAs), are transcribed from approximately 98% of the genomic loci in eukaryotic cells. As a widespread RNA species in eukaryotic cells, circRNAs present covalently closed continuous loop structures from the covalently joined 3′- and 5′-ends of a single-stranded RNA molecule (termed as backsplicing) during RNA splicing, without 5′–3′ polarity and a polyadenylated poly(A) tail [5]. Owing to the lack of accessible ends, circRNAs are more stable than linear RNAs and can survive exonuclease RNase R, which possesses strong 3′-exonuclease activity for linear or Y-shaped RNA degradation [6]. CircRNAs arise from exons (exonic circRNAs), introns (intronic circRNAs), or a combination of both (exon–intron circRNAs), and the subcellular localization of the three types of circRNAs often shows different compartment preferences [7]. Moreover, circRNAs are usually expressed in a developmental stage-, tissue-, and cell-specific manner [8–10]. Such diverse subcellular localizations and expression patterns suggest the complexity of the functions and regulatory mechanisms of circRNAs. General mechanisms of circRNA functions include competing endogenous RNAs (ceRNAs) that sponge miRNAs, interacting with RNA-binding protein, protein scaffolding, enhancer of protein function for gene transcription activation, competition with precursor mRNA splicing, and templates for translation to produce unique circRNA peptides [7, 11]. Although circRNAs were previously considered as the transcriptional "noise" generated by aberrant splicing events, with the development of high-throughput sequencing and continuous exploration of their diverse functions, circRNAs have been shown to play vital roles in growth and development, metabolic processes, diseases, and cancer. CircRNAs have been extensively proposed to serve as highly effective biomarkers in obesity and disease diagnosis, treatment, and prognosis, and thus have become the latest research hotspot in the RNA field [12].

Increasing evidence has revealed a few circRNAs that are functionally and mechanistically characterized in mammalian adipogenesis, such as circTshz2-1 and circArhgap5-2 [13], circSAMD4A [14], circFOXP1 [15], CDR1as [16], circH19 [17], circFLT1 [18], sus_circPAPPA2 [19], and circPPARA [20]. Intriguingly, recent studies have shown that mammalian adipocyte-expressed circRNAs also affect various other organs, including the liver and skin, via exosome, a crucial carrier mediating signal conduction between adipocytes and target organs, resulting in the transcriptomic evaluation of target organs such as circ-DB [21], circ_0075932 [22], and mmu_circ_0000623 [23]. In chickens, extremely limited investigations have been conducted on the precise roles and molecular mechanisms of circRNAs in adipogenesis, although the expressed circRNAs in abdominal adipose tissue have been authenticated. A total of 275 differentially expressed circRNAs, whose parental genes are significantly involved in lipid-related biological processes and signaling pathways, have been identified in the abdominal fat tissue of Chinese domestic Gushi chickens at 6, 14, 22, and 30 weeks of age. The ceRNA network indicated that these differentially expressed circRNAs might play endogenous competitive regulatory roles through interactions with multiple miRNAs during abdominal fat tissue development in chickens [24]. Integrative analysis of circRNA, miRNA, and mRNAs profiles during chicken intramuscular and abdominal adipogenic differentiation revealed that circLCLAT1, circFNDC3AL, circCLEC19A, and circARMH1 potentially affect intramuscular and abdominal adipogenesis by sponging miRNAs to regulate downstream genes involved in peroxisome proliferator-activated receptor (PPAR)- and fatty acid metabolism-related signaling pathways [25]. A comparative analysis of circRNA expression in abdominal fat tissues with high and low abdominal fat percentage of Guangxi Partridge chickens identified 26 differentially expressed circRNAs. Of these, novel_circ_PTPN2, novel_circ_CTNNA1, and novel_circ_PTPRD may serve as ceRNAs to regulate abdominal fat deposition [26]. In our previous study, we identified 111 differentially expressed circRNAs in chicken abdominal adipocytes at 0, 12, 48, 72, and 120 h post-differentiation, wherein Z:35,565,770|35,568,133 and Z:54,674,624|54,755,962 were considered candidate regulators of chicken adipogenic differentiation. Z:35,565,770|35,568,133 may compete with precursor mRNA splicing of its lipogenesis-related parental gene, abhydrolase domain containing 17B, depalmitoylase (*ABHD17B*), and Z:54,674,624|54,755,962 may function as a ceRNA that competitively sponges miRNAs to regulate the expression of lipogenesis-related genes [27]. These results imply that circRNAs might function as pivotal regulators of adipogenesis and abdominal fat deposition in chickens. Therefore, further studies are needed to reveal the multifaceted roles of circRNAs in adipogenesis and uncover the underlying molecular mechanisms in chickens.

In the present study, we identified a gradually increasing and differentially expressed circRNA, 8:27,886,180|27,889,657, based on our previously reported circRNA sequencing data during chicken abdominal preadipocyte adipogenic differentiation (accession number: PRJNA732104) [27]. The circRNA 8:27,886,180|27,889,657 is composed of exons 19–22 of the linear transcript of the dedicator of cytokinesis 7 (*DOCK7*) gene with a length of 654 nucleotides (abbreviated as circDOCK7). We then explored its characteristics, expression patterns, biological functions, and regulatory mechanisms, and found that circDOCK7 could promote the proliferation and adipogenic differentiation of chicken abdominal preadipocytes by serving as a miRNA sponge to directly sequester gga-miR-301b-3p away from the acyl-CoA synthetase long-chain family member 1 (*ACSL1*) gene and promote its expression post-transcriptionally. To our best knowledge, our study is the first to elucidate the regulatory mechanism of circRNA-mediated adipogenesis in chickens, providing new insights into the regulation of avian adipogenesis and a promising selective biomarker for the genetic improvement of excessive abdominal fat deposition in chickens.

## Methods

### Ethics statement

All animal experiments were conducted in accordance with the protocols approved by the Animal Welfare Committee of the State Key Laboratory for Agro-Biotechnology of the China Agricultural University (Permit Number: XK257). The birds were raised under the same environmental conditions, and food and water were provided ad libitum. For animal welfare, the birds were humanely slaughtered, and all efforts were made to minimize suffering.

### Experimental animals and sample preparation

A population of 42-day-old female Arbor Acres broilers was humanely slaughtered to collect abdominal fat. According to abdominal fat percentage (AbFP, abdominal fat weight/eviscerated weight), eight broilers each with extremely high AbFP (HAbF, 1.83% ± 0.23%) and with extremely low AbFP (LAbF, 0.43% ± 0.24%), which showed an extremely significant statistical difference of AbFP between HAbF group and LAbF group [28], were selected to collect the abdominal adipose tissue (leaf fat), liver, heart, pancreas, kidney, pectoralis, and duodenum. These tissue samples were immediately snap-frozen in liquid nitrogen and stored at −80 ºC for further RNA extraction.

### Cell culture and adipogenic differentiation

Immortalized chicken preadipocyte 2 (ICP2) cells were obtained from the Key Laboratory of Chicken Genetics and Breeding, Ministry of Agriculture (Northeast Agricultural University, Harbin, Heilongjiang, China). The ICP2 cells were maintained in a basal medium consisting of Dulbecco’s modified Eagle’s medium F12 (DMEM-F12) (Gibco, Gaithersburg, MD, USA) with a glucose concentration of 17.51 mmol/L supplemented with 10% fetal bovine serum (FBS) (Gibco) and 1% penicillin–streptomycin (Gibco). While growing to 80% confluence, ICP2 cells were incubated with differentiation medium comprising the basal medium supplemented with a final concentration of 160 μmol/L sodium oleate (Sigma, St. Louis, MO, USA) dissolved in sterile deionized water to induce adipogenic differentiation. The differentiation medium was changed every day. Human embryonic kidney 293 T cells and chicken embryonic fibroblast DF1 cells were obtained from the American Type Culture Collection (ATCC, Manassas, VA, USA) and were maintained in DMEM (Gibco) supplemented with 10% FBS (Gibco) and 1% penicillin–streptomycin (Gibco). All cells were cultured at 37 °C in a humidified 5% CO_2_ atmosphere.

### Complementary DNA (cDNA) synthesis and quantitative real-time PCR (qRT-PCR)

ICP2 cells were lysed using TRIzol reagent (TIANGEN, Beijing, China) for total RNA extraction. To detect circDOCK7 and mRNA expression, 2 μg of total RNA from each sample was reverse-transcribed into cDNA using the FastKing RT Kit (with gDNase) (TIANGEN) according to the manufacturer’s recommendations. A SYBR Green-based qRT-PCR was performed in a 20-μL reaction volume containing 10 μL 2× Universal SYBR Green Fast qPCR Mix (aBclonal, Wuhan, China), 8 μL RNase-free water, 0.5 μL each of forward and reverse primers (10 μmol/L), and 1 μL cDNA (approximately 300 ng). The qRT-PCR amplification protocol consisted of an initial denaturation at 95 °C for 15 min, 40 cycles of denaturation at 94 °C for 20 s as well as annealing and extension at 60 °C for 34 s. The housekeeping gene *GAPDH* served as an internal control to normalize the relative circRNA and mRNA expression.

To test miRNA expression, 2 μg of total RNA from each sample was reverse-transcribed into cDNA using the miRcute Plus miRNA First-Strand cDNA Kit (TIANGEN). A SYBR Green-based poly(A)-tailed qRT-PCR was performed in a 20-μL reaction volume containing 10 μL 2× miRcute Plus miRNA PreMix (SYBR&ROX) (TIANGEN), 8.2 μL RNase-free water, 0.4 μL each of forward and reverse primers (10 μmol/L), and 1 μL cDNA (approximately 300 ng). The miRNA qRT-PCR amplification protocol consisted of an initial denaturation at 95 °C for 15 min, 5 cycles of denaturation at 94 °C for 20 s, annealing at 64 °C for 30 s, and extension at 72 °C for 34 s, followed by 40 cycles of 94 °C for 20 s, 60 °C for 34 s. The housekeeping gene *U6* served as an internal control to normalize relative miRNA expression. The qRT-PCR was performed on a Bio-Rad CFX96 Real-Time PCR system (Bio-Rad Laboratories, Inc., Hercules, CA, USA). The relative expression levels of circDOCK7, miRNAs and mRNAs were calculated using the 2^−∆∆CT^ method. The qRT-PCR primers for miRNA expression were designed using the miRprimer2 software [29] and those for circRNA and mRNA expression were designed using the National Center for Biotechnology Information (NCBI) Primer-BLAST [30]. All primers were synthesized by SinoGenoMax (Beijing, China) (Additional File 1: Table S1).

### Cyclization validation of circDOCK7

Divergent and convergent primers were designed to amplify the backsplicing junction (BSJ) sites of circDOCK7 or linear *DOCK7* mRNA, using cDNA and genomic DNA (gDNA) templates from chicken abdominal fat, respectively. The back-splicing sites of circDOCK7 were PCR-amplified by divergent primer PCR and validated by Sanger sequencing. To compare the half-lives of circDOCK7 and *DOCK7*, ICP2 cells were treated with a final dose of 2 μg/mL actinomycin D (Acmec, Shanghai, China) for 0, 4, 8, 12 and 24 h and then collected for further RNA extraction and expression analysis as mentioned above. To validate circDOCK7 resistance to RNase R digestion, 4 μg RNA from ICP2 cells was treated with RNase R in three replicates (20 U/μL, GENESEED, Guangzhou, Guangdong, China) according to the manufacturer’s instructions. For the control group, 4 μg of RNA was supplemented with RNase-free water and 10× RNase R buffer (RNase R−). For the RNase R digestion group, 4 μg RNA was mixed with 10 U RNase R, RNase-free water, and 10× RNase R buffer (RNase R+). Subsequently, the two groups were incubated for 30 min at 37 °C. The RNA samples were reverse-transcribed into cDNA using the FastKing RT Kit (with gDNase) (TIANGEN). The expression of circDOCK7 and *DOCK7* in the two groups was detected by qRT-PCR. *GAPDH* was used as an internal control.

### Isolation of nuclear and cytoplasmic fractions

The nuclear and cytoplasmic fractions and total RNA from ICP2 cells were isolated using a PairsTM kit (Part Number AM1921, Invitrogen, Carlsbad, CA, USA) according to the manufacturer’s instructions. cDNA was synthesized as previously described. The *U6* and *GAPDH* genes were used as marker genes for nuclear and cytoplasmic RNAs, respectively.

### Fluorescence in situ hybridization (FISH)

A 5′-FAM-labeled probe 5′-TTCAT + TCCAGGAAGGGGAACC + TTACTGCCAATGATGGA-3′ specifically binding to circDOCK7 was designed and synthesized by GenePharma Co., Ltd. (Shanghai, China). ICP2 cells were seeded in confocal culture dishes and were fixed with 4% paraformaldehyde (Solarbio, Beijing, China). The fixed ICP2 cells were then hybridized with 2 μmol/L of fluorescence probes using a Fluorescent In Situ Hybridization Kit (Genepharma, Shanghai, China) according to the manufacturer’s instructions. The 4′,6-diamidino-2-phenylindole (DAPI) (Solarbio) was used to stain nuclei. Images were captured using a laser scanning confocal microscope (Nikon A1R, Tokyo, Japan).

Bioactive mitochondria were specifically labeled using MitoTracker Red CMXRos (Beyotime, Shanghai, China) in ICP2 cells, according to the manufacturer’s instructions. Briefly, the cells were cultured in a confocal dish. After removing the basal medium, a final concentration of 100 nmol/L MitoTracker Red CMXRos diluted with Hank’s Balanced Salt Solution (HBSS, with Ca^2+^ and Mg^2+^) (Beyotime) was added and incubated at 37 ºC for 30 min. Thereafter, the MitoTracker Red CMXRos was replaced with pre-heated fresh basal medium at 37 ºC. The endoplasmic reticulum was specifically labeled with ER-Tracker Red (Beyotime) in ICP2 cells, according to the manufacturer’s instructions. Briefly, cells were cultured in a confocal dish and washed with HBSS (Beyotime). Subsequently, the cells were incubated with pre-heated 1:1,000 ER-Tracker Red staining solution of ER-Tracker Red and ER-Tracker Red diluent at 37 °C for 30 min. ICP2 cells were observed microscopically using a laser scanning confocal microscope (Nikon A1R).

### Cell Counting Kit-8 (CCK-8) assay

ICP2 cells were seeded in 96-well plates and cultured in basal medium. At 6, 24, 48, 72 and 96 h post-transfection, cell proliferation was monitored using the Cell Counting Kit-8 (Beyotime), according to the manufacturer’s protocol. After 1 h of incubation, absorbance at 450 nm was measured using a SpectraMax® i3x Multi-Mode Microplate Reader (Molecular Devices Corporation, Sunnyvale, CA, USA).

### 5-Ethynyl‐2′‐deoxyuridine (EdU) assay

ICP2 cells were seeded in 12-well plates and cultured in basal medium. At 48 h post-transfection, the cells were stained with Alexa Fluor 555 for 2 h using a BeyoClick™ EdU Cell Proliferation Kit (Beyotime), according to the manufacturer’s protocol. Cell nuclei were stained blue and EdU-positive cells were stained red. Cells were microscopically observed and photographed using an Echo Revolve fluorescence microscope (Echo Laboratories, San Diego, CA, USA). The EdU-positive and total cells in the photofluorogram were counted using the ImageJ software [31].

### Cell cycle assay

ICP2 cells were seeded in 6-well plates and cultured in basal medium. At 48 h post-transfection, cells were harvested by centrifugation at 1,000 × *g* for 5 min. After washing with cold phosphate buffered saline (PBS) (Gibco), the cells were fixed in cold 70% ethanol at 4 ℃ overnight. Subsequently, the cells were re-collected by centrifugation at 1,000 × *g* for 5 min and incubated with propidium staining solution (Beyotime) at 37 ºC for 30 min in the dark conditions, according to manufacturer’s instructions. The cell cycle was analyzed by flow cytometry using a BD FACSCalibur flow cytometer (BD Biosciences, San Diego, CA, USA), and the data were analyzed using the FlowJo_v10.8.1 software.

### Measurement of intracellular acetyl coenzyme A (acetyl-CoA) and triglycerides (TG)

To investigate the effects of genes on intracellular acetyl-CoA and triglycerides, ICP2 cells were treated with a final concentration of 160 μmol/L sodium oleate (Sigma) for 48 h after transfection. ICP2 cells were then washed twice with PBS and lysed with the lysis buffer. After ultrasonication, the cell mixture was resuspended by centrifugation at 8,000 × *g* for 10 min at 4 ºC. The intracellular acetyl-CoA content was determined using an Acetyl Coenzyme A Content Assay Kit (Boxbio, Beijing, China), according to the manufacturer’s instructions. The ICP2 cells were then washed twice with PBS, and lysis buffer was added for ultrasonication. Thereafter, the cell suspension was incubated for 10 min at 70 °C and centrifuged at 2,000 × *g* for 5 min at 25 °C. The intracellular triglyceride content was detected using a Tissue Triglyceride (TG) Content Assay Kit (Applygen, Beijing, China), according to the manufacturer’s instructions. The intracellular total protein content was assessed using a BCA Protein Quantification Kit (Applygen) to normalize the acetyl-CoA and triglyceride content.

### Glucose uptake capacity assay

ICP2 cells were seeded into 6-well plates and transfected with the pcDNA3.1-EGFP and pcDNA3.1-ACSL1-EGFP plasmids. At 48 h post-transfection, the glucose uptake capacity of the cells was monitored using the Glucose Uptake Assay Kit (Dojindo, Tokyo, Japan), according to the manufacturer’s instructions. Briefly, the cells were washed twice and incubated with pre-heated glucose-free medium (Solarbio) for 15 min at 37 °C. Thereafter, the cells were incubated with the pre-heated probe solution for 15 min at 37 ºC and washed twice with pre-cooled WI Solution (1×). Pre-cooled WI Solution (1×) was added again and incubated at 25 °C for 5 min. Cells were microscopically observed and photographed using an Echo Revolve fluorescence microscope (Echo Laboratories). The total number of cells was determined using the Cell Count Normalization Kit (Dojindo), according to the manufacturer’s instructions. The fluorescence intensity of the cell nucleus (excitation filter: 350 nm; emission filter: 461 nm) and glucose uptake (excitation filter: 545 nm; emission filter: 605 nm) were measured using a SpectraMax® i3x Multi-Mode Microplate Reader (Molecular Devices Corporation).

### Oil Red O staining

ICP2 cells were washed thrice with PBS (Gibco) and fixed in 4% paraformaldehyde solution (Solarbio) for 30 min. After washing thrice again with PBS, the cells were stained with Oil Red O (Sigma) dissolved in 100% isopropyl alcohol for 15 min at 25 ºC. Following infiltration with 60% isopropyl alcohol for 10 s, the cells were washed thrice with PBS and imaged under a microscope (Leica, Bensheim, Germany). Intracellular Oil Red O was dissolved in 100% isopropyl alcohol for 5 min, and the content of lipid droplets was quantified spectrophotometrically by measuring the absorbance at 490 nm.

### Nile red fluorescent staining

ICP2 cells were washed thrice with PBS, fixed in 4% formaldehyde (Solarbio) for 10 min, and then incubated with Nile red fluorescent dye (Applygen) for 10 min at 25 ºC. After washing with PBS, the cells were incubated with DAPI for 10 min to stain the nuclei at 25 ºC. Cells were microscopically observed and photographed using an Echo Revolve fluorescence microscope (Echo Laboratories).

### Bioinformatics analysis

The secondary structure of circDOCK7 was predicted using the RNAfold web server [32]. Open reading fragments (ORFs) with a minimum length of 150 nt were extracted using the EMBOSS GetOrf software [33]. The internal ribosome entry site (IRES) elements of circDOCK7 were predicted using IRESfinder, a specialized tool for IRES prediction in eukaryotic cells [34]. The N6-methyladenosine (m^6^A) modification sites in the circDOCK7 sequence were predicted using SRAMP (sequence-based RNA adenosine methylation site predictor) [35]. The RNA-RNA duplex and minimum free energy (MFE) between circDOCK7 and gga-miR-301b-3p as well as between gga-miR-301b-3p and the 3′ UTR of the *ACSL1* gene were analyzed using RNAhybrid [36]. Subcellular localization of chicken ACSL1 protein were predicted using UniProt [37].

### Construction of the ceRNA regulatory network

Differentially expressed miRNAs that potentially interact with circDOCK7 and their differentially expressed target mRNAs that are closely involved in lipid metabolism were predicted using an intersection of miRanda [38] and TargetScan [39]. The circDOCK7-miRNA pairs with a negative expression correlation, miRNA-mRNA pairs with a negative expression correlation, and circDOCK7-mRNA pairs with a positive expression correlation were selected to establish the circDOCK7-associated ceRNA network, which was visualized using the Cytoscape software, version 3.8.2 [40].

### Plasmid construction and RNA oligonucleotide synthesis

To construct dual-luciferase reporter plasmids, the *ACSL1* 3′ UTR region containing the complementary binding sites of the gga-miR-301b-3p seed region with an insert containing XhoI and NotI restriction enzyme sites was cloned into the XhoI and NotI (Takara, Tokyo, Japan) double-digested psi-CHECK™-2 vector (Promega, Madison, WI, USA) using the Trelief™ SoSoo Cloning Kit (TSINGKE, Beijing, China) for wild-type plasmid construction, and named ACSL1-3′UTR-WT, according to the manufacturer's specifications. The complementary binding site of gga-miR-301b-3p to the *ACSL1* 3′-UTR was deleted and cloned to construct the mutant-type plasmid ACSL1-3′-UTR-Mut. Similarly, the wild-type circDOCK7 plasmid containing a complementary gga-miR-301b-3p binding site (circDOCK7-WT) and the mutant-type circDOCK7 plasmid of lacking the complementary gga-miR-301b-3p binding site (circDOCK7-Mut) were constructed. All primers were synthesized by SinoGenoMax (Beijing, China) (Additional File 1: Table S1).

To construct the circDOCK7 overexpression vector, the full-length circDOCK7 with EcoRI and BamHI restriction enzyme sites was cloned into the EcoRI and BamHI (Takara) double-digested pLC5-ciR vector (GENESEED, Guangzhou, China) using the Trelief™ SoSoo Cloning Kit (TSINGKE), and named oe-circDOCK7. To construct the *ACSL1* overexpression vector, the complete coding sequences of the *ACSL1* gene with a deleted termination codon and inserted NheI and EcoRI restriction enzyme sites were cloned into the NheI and EcoRI (Takara) double-digested pcDNA3.1-EGFP vector (Invitrogen), using the Trelief™ SoSoo Cloning Kit (TSINGKE), and named pcDNA3.1-ACSL1-EGFP. Plasmid DNA was purified using the EndoFree Maxi Plasmid Kit (TIANGEN), according to the manufacturer’s instructions.

Small interfering RNA (siRNA) oligonucleotides siACSL1 (sense: CCUCACGACCUACUGGUAUTT; antisense: AUACCAGUAGGUCGUGAGGTT) designed to specifically knockdown the *ACSL1* gene, the siRNA oligonucleotides sicircDOCK7 (sense: CAUUGGCAGUAAGGUUCCCTT; antisense: GGGAACCUUACUGCCAAUGTT) designed to specifically knockdown circDOCK7, miR-301b-3p agomir specifically for gga-miR-301b-3p overexpression, miR-301b-3p antagomir specifically for gga-miR-301b-3p knockdown, and their corresponding negative controls (NCs), were synthesized by GenePharma Co., Ltd. (Shanghai, China). All cell transfection experiments were performed using Lipofectamine 3000 Transfection Reagent (Invitrogen), according to the manufacturer’s instructions.

### Dual-luciferase reporter assay

After seeding 293 T cells and DF1 cells in 24-well plates, they were co-transfected in triplicate with miR-301b-3p agomir or agomir NC (80 nmol/L) and the above-mentioned wild-type or mutant plasmids (500 ng per well). At 48 h post-transfection, the cells were washed three times with PBS and lysed with 1× passive lysis buffer (Promega) for 15 min. Firefly and *Renilla* luminescence were measured using the Dual-Luciferase® Reporter Assay System (Promega) on a SpectraMax® i3x Multi-Mode Microplate Reader (Molecular Devices Corporation). *Renilla* luciferase activity was normalized to the firefly luciferase activity.

### RNA Pull-down assay

A biotinylated probe specifically binding to circDOCK7 was designed, and an oligo probe served as a negative control (NC). Approximately, 1 × 10^7^ ICP2 cells were harvested, lysed, and ultrasonicated. The biotinylated circDOCK7 probe (TSINGKE) and NC probe were denatured by incubating at 90 °C for 2 min and then in an ice bath for 2 min. After adding 50 μL of RNA structure buffer and 20 μL of RNase-free water, the probes were kept at 25 °C for 20 min to form the RNA secondary structure. Subsequently, the probes were incubated with streptavidin magnetic beads (Life Technologies, Grand Island, NY, USA) at 25 ºC for 30 min to generate probe-coated beads. The cell lysates were incubated with probe-coated beads at 4 °C overnight. The miRNAs that were sponged by circDOCK7 in the pull-down materials were reverse-transcribed into cDNA, and gga-miR-301b-3p expression was analyzed by a qRT-PCR assay, as mentioned above.

### Statistical analysis

All data are presented as mean ± standard deviation. The relative gene expression levels are calculated using 2^−∆∆Ct^ method and shown as fold changes compared with that in the control group. The normality of experimental data was performed using Shapiro–Wilk test before the significant difference by statistics analysis. Statistically significant differences between the two experimental groups were determined via the *t*-test using the SPSS software (version 23.0; IBM, Chicago, IL, USA). Significance was set at *P* < 0.05, and extreme significance was set at *P* < 0.01. The results were analyzed using GraphPad Prism 8 (GraphPad Software, San Diego, CA, USA).

## Results

### Characterization and expression profile of circDOCK7

**Fig. 1:**
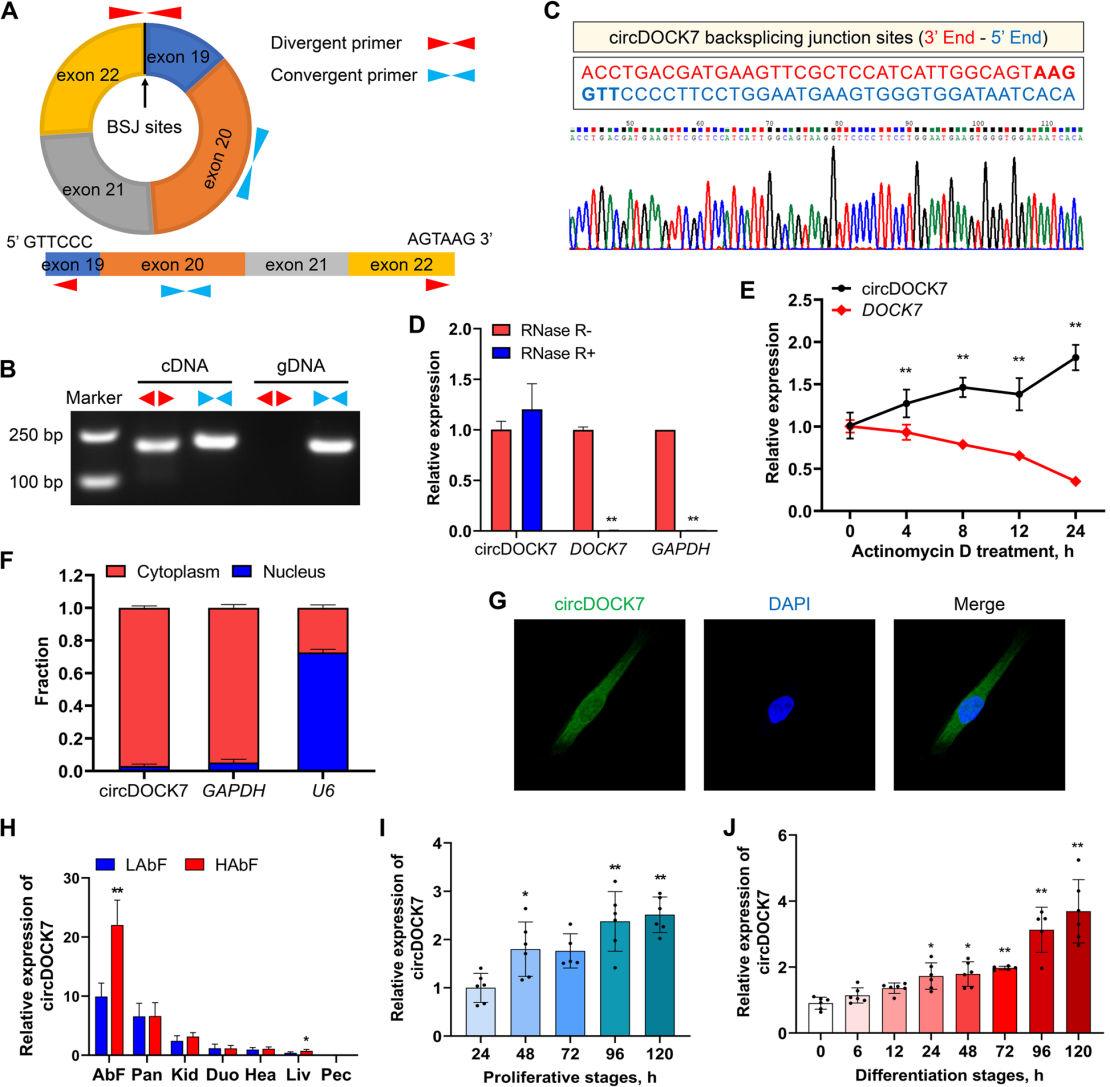
Cyclization verification, subcellular localization, and expression pattern analysis of circDOCK7. **A** Schematic diagram showing the formation and designing of primers for cyclization verification of circDOCK7. **B** PCR amplification of the divergent and convergent primers of circDOCK7 using gDNA and cDNA as templates, respectively. **C** Confirmation of the backsplicing junction of circDOCK7 via Sanger’s sequencing. **D** qRT-PCR analysis of the relative expression of circDOCK7 and *DOCK7* in chicken abdominal preadipocytes treated with RNase R. The relative gene expression levels are shown as fold changes compared with that in the RNase R − group. **E** qRT-PCR analysis of the relative expression of circDOCK7 and *DOCK7* in chicken abdominal preadipocytes treated with actinomycin D. The relative gene expression levels are shown as fold changes compared with that in the 0 h group. **F** qRT-PCR analysis of circDOCK7 expression in the cytoplasm and nuclear fractions of chicken abdominal preadipocytes. **G** Subcellular localization of circDOCK7 in chicken abdominal preadipocytes using RNA fluorescence in situ hybridization assay. Cell nuclei were stained with 4′,6-diamidino-2-phenylindole DAPI (blue), and circDOCK7 was hybridized with the circDOCK7 probe (green). **H** Tissue expression pattern of circDOCK7 in chickens with high and low abdominal fat percentage. **I **and** J** Expression pattern of circDOCK7 during the proliferation and adipogenic differentiation of chicken abdominal preadipocytes. During the proliferation of chicken abdominal preadipocytes, the relative expression levels are shown as fold change versus that of the 24 h group, and the statistical significance analysis are performed versus the 24 h group as control. During the adipogenic differentiation of chicken abdominal preadipocytes, the expression levels are shown as fold change versus that of the 0 h group, and the statistical significance analysis are performed versus the 0 h group as control.  ^*^P < 0.05, ^**^P < 0.01. The same below

circDOCK7 is a 654-nucleotide-long circRNA derived from the head-to-tail splicing of exons 19–22 of the *DOCK7* gene located on chromosome 8 (Fig. 1A, Additional File 1: Table S2). To exclude the possibility that such head-to-tail splicing products arise from genomic rearrangements and trans-splicing, we first designed divergent primers that specifically amplify only the circular transcript (circDOCK7) and convergent primers that specifically amplify the linear parental transcript (*DOCK7*) using cDNA and gDNA templates, respectively (Fig. 1A). The circDOCK7 was observed only in divergent primer-amplified PCR products from cDNA, but not from the gDNA of ICP2 cells, and the backsplicing junction sites were validated by Sanger sequencing (Fig. 1B and C). Moreover, endogenous circDOCK7 abundance was not significantly changed upon exposure to RNase R, in which the significantly decreased expression levels of the linear *DOCK7* and *GAPDH* genes supported the desirable efficacy of the RNase R treatment, indicating that circDOCK7 was resistant to RNase R digestion (Fig. 1D). The RNA stability of circDOCK7 and its linear parental gene *DOCK7*, as determined using actinomycin D, a small molecule inhibitor of transcription, showed that circDOCK7 was highly stable with its half-life exceeding 24 h, while the expression of the *DOCK7* gene was remarkably reduced in a time-dependent manner and exhibited more than 65% reduction at 24 h (Fig. 1E). These results suggest that circDOCK7 was stable with RNase R resistance and a longer half-life. The secondary structure prediction of circDOCK7, based on the minimum free energy (MFE), showed that circDOCK7 has a stem-loop structure (Additional file 2: Fig. S1). To determine the subcellular localization of circDOCK7, we conducted a qRT-PCR analysis of nuclear and cytoplasmic circDOCK7 expression and FISH with a 5'FAM-labeled circRNA probe that precisely recognizes the backsplicing junction sites of circDOCK7 backsplicing region to determine its subcellular location. We found that circDOCK7 was primarily located in the cytoplasm of ICP2 cells (Fig. 1F and G). These results indicated that circDOCK7 is an exon-based RNase-resistant circRNA that predominantly localizes to the cytoplasm.

The expression profiles determined by qRT-PCR showed that circDOCK7 had the highest expression in abdominal fat among the seven detected tissues, and a significantly higher expression in abdominal fat tissues in the high abdominal fat (HAbF) group than in the low abdominal fat (LAbF) group (Fig. 1H). During the proliferation and adipogenic differentiation of chicken abdominal preadipocytes, the expression of circDOCK7 gradually increased in a time-dependent manner (Fig. 1I and J). These findings indicate that circDOCK7 may play a positive regulatory role in adipogenesis.

### CircDOCK7 knockdown inhibits the proliferation of chicken abdominal preadipocytes

**Fig. 2:**
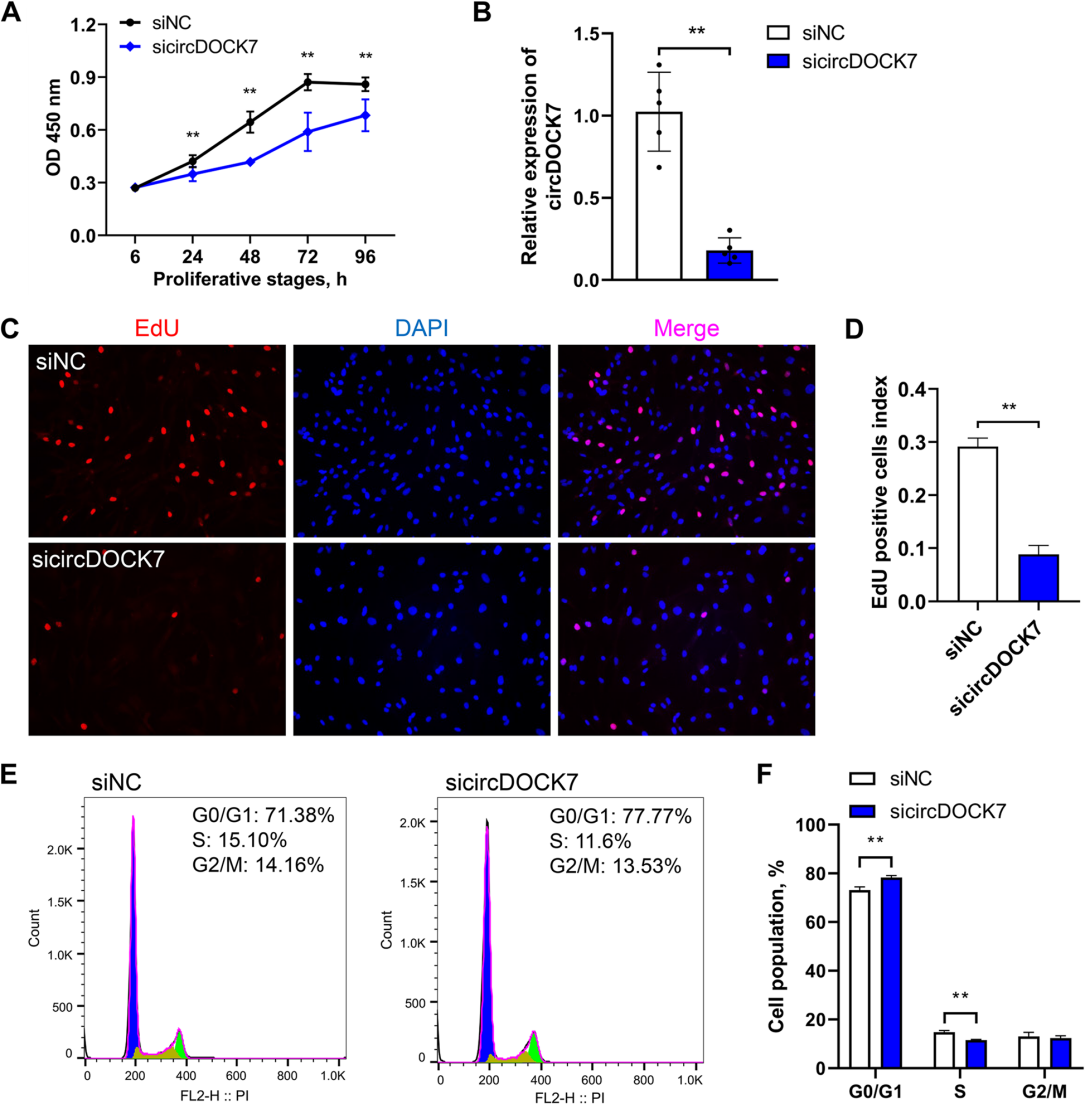
Effects of circDOCK7 knockdown on the proliferation of chicken abdominal preadipocytes. **A** CCK8 assay of chicken abdominal preadipocytes transfected with siNC and sicircDOCK7 at 6, 24, 48, 72 and 96 h post-transfection. **B** Detection of circDOCK7 knockdown in chicken abdominal preadipocytes 48 h post-transfection with sicircDOCK7. The relative gene expression levels are shown as fold changes compared with that in the siNC group. The same below. **C** Proliferation of chicken abdominal preadipocytes determined by 5-ethynyl-2′-deoxyuridine (EdU) after 48 h of transfection with sicircDOCK7. **D** Histogram showing the proportion of EdU-positive cells using ImageJ. **E** and **F** Cell cycle analysis of chicken abdominal preadipocytes after 48 h transfection with sicircDOCK7 using flow cytometry. ^*^P < 0.05, ^**^P < 0.01

To explore the effects of circDOCK7 on abdominal adipogenesis in chickens, we first monitored the cell proliferation capacity of chicken abdominal preadipocytes via gain- and loss-of-function experiments by transfecting the circDOCK7 overexpression vector pLC5-circDOCK7 and siRNA sicircDOCK7, respectively. The CCK8 assay showed no significant difference in the number of living chicken abdominal preadipocytes in the experimental group compared to the control group at 6, 24, 48, 72 and 96 h post-transfection (Additional file 2: Fig. S2A). The remarkable overexpression of circDOCK7 (by approximately 2,700-fold) in pLC5-circDOCK7-treated ICP2 cells for 48 h was confirmed by qRT-PCR (Additional file 2: Fig. S2B). The EdU staining showed no significant change in the proportion of EdU-positive chicken abdominal preadipocytes in the circDOCK7 overexpression group compared to the control group (Additional file 2: Fig. S2C and D). A flow-cytometric cell cycle analysis revealed that circDOCK7 overexpression did not significantly change the G0/G1-, S-, and G2/M-phase populations of chicken abdominal preadipocytes (Additional file 2: Fig. S2E and F). CircDOCK7 knockdown significantly decreased the number of living chicken abdominal preadipocytes, as determined by the CCK8 assay (Fig. 2A). A qRT-PCR analysis showed that circDOCK7 expression caused an approximately 84% reduction in sicircDOCK7-treated ICP2 cells for 48 h (Fig. 2B). The EdU staining assay revealed significantly reduced EdU-positive chicken abdominal preadipocytes in the circDOCK7 knockdown group compared to the siNC group (Fig. 2C and D). Moreover, circDOCK7 knockdown prevented cell cycle progression, resulting in more cell populations arrested in the G0/G1 phase and, thus, fewer cell populations in the S phase of chicken abdominal preadipocytes (Fig. 2E and F), indicating that circDOCK7 knockdown inhibited the proliferation of chicken abdominal preadipocytes.

### CircDOCK7 promotes the adipogenic differentiation of chicken abdominal preadipocytes

**Fig. 3:**
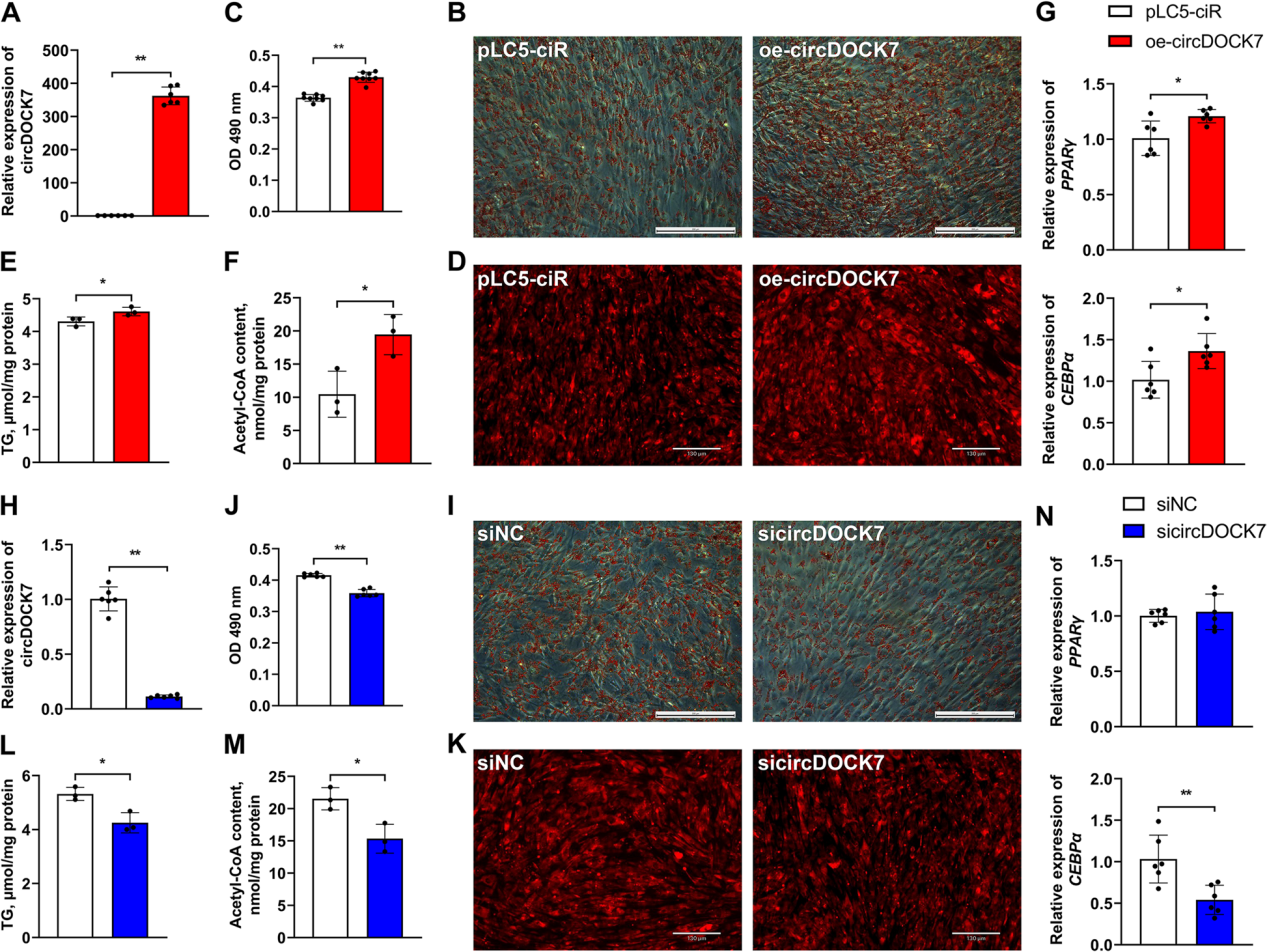
Effects of circDOCK7 on the adipogenic differentiation of chicken abdominal preadipocytes. **A** Detection of circDOCK7 overexpression after 48 h of transfection with circDOCK7 overexpression vector in differentiated chicken abdominal adipocytes. The relative gene expression levels are shown as fold changes compared with that in the pLC5-ciR group. The same below. **B** and **C** Representative images of Oil Red O staining and lipid droplet content of circDOCK7-overexpressing differentiated chicken abdominal adipocytes. **D** Representative images of Nile red fluorescent staining of circDOCK7-overexpressing differentiated chicken abdominal adipocytes. **E **and **F** Intracellular triglyceride and acetyl-CoA levels of circDOCK7-overexpressing differentiated chicken abdominal adipocytes. **G** Relative mRNA expression levels of *PPARγ* and *CEBPα* in circDOCK7-overexpressing differentiated chicken abdominal adipocytes. **H** Detection of circDOCK7 knockdown after 48 h of transfection with sicircDOCK7 in differentiated chicken abdominal adipocytes.** I** and** J** Representative images of Oil Red O staining and lipid droplet content of circDOCK7-knockdown differentiated chicken abdominal adipocytes. **K** Representative images of Nile red fluorescent staining of circDOCK7-knockdown differentiated chicken abdominal adipocytes. **L** and **M** Intracellular triglyceride and acetyl-CoA levels of circDOCK7-knockdown differentiated chicken abdominal adipocytes. **N** Relative mRNA expression levels of *PPARγ* and *CEBPα* in circDOCK7-knockdown differentiated chicken abdominal adipocytes. ^*^P < 0.05, ^**^P < 0.01

To further investigate the regulatory role of circDOCK7 in abdominal adipogenesis in chickens, we detected intracellular lipid droplet accumulation and intracellular triglyceride and acetyl-CoA contents via gain- and loss-of-function assays in differentiated ICP2 cells. Compared with the pLC5-ciR control group, a remarkable increase in circDOCK7 expression (by approximately 360-fold) was detected in circDOCK7-overexpressing ICP2 cells by a qRT-PCR analysis (Fig. 3A). This resulted in a significant increase in intracellular lipid droplet accumulation upon circDOCK7 overexpression, as determined by Oil Red O and lipophilic fluorophore Nile red staining (Fig. 3B–D). Moreover, excessive circDOCK7 expression led to a significant increase in the content of intracellular triglycerides and acetyl-CoA, the main precursor of lipid synthesis (Fig. 3E and F). The mRNA expression of marker genes closely responsible for adipogenesis, including peroxisome proliferator-activated receptor γ (*PPARγ*) and CCAAT/enhancer-binding protein *α* (*CEBPα*), was significantly increased upon circDOCK7 stimulation (Fig. 3G). Conversely, intracellular lipid droplet accumulation as well as acetyl-CoA and triglyceride levels were remarkably decreased by circDOCK7 interference, accompanied by significantly inhibited mRNA abundance of the *CEBPα* gene (Fig. 3H–N). These results indicate that circDOCK7 facilitates the adipogenic differentiation of chicken abdominal preadipocytes.

### CircDOCK7 acts as a sponge for gga-miR-301b-3p to play biological roles

circRNAs are implicated in various biological processes through diverse regulatory mechanisms, such as sponging miRNA, modulating their host gene transcription, and encoding proteins or peptides. To elucidate the functional mechanism of circDOCK7 underlying adipogenesis in chickens, we first evaluated the translation potential of circDOCK7 based on the fact that endogenous circRNAs with ORFs can be translated into proteins or peptides driven by an IRES and m^6^A modification. A bioinformatics analysis revealed that circDOCK7 has an IRES (Score 0.7133933991446173) and two m^6^A modification sites in 195A (high confidence) and 369A (moderate confidence) (Additional file 2: Fig. S3), but no ORFs, suggesting that it has almost no protein-coding potential. Second, we assessed the co-expression tendency of circDOCK7 and *DOCK7* during adipogenic differentiation of chicken abdominal preadipocytes and found that circDOCK7 had no significant correlation with its parental genes (Pearson correlation coefficient =  −0.3024, *P* = 0.2734) (Additional file 2: Fig. S4A and B). To further confirm whether circDOCK7 modulated the transcriptional expression of its host *DOCK7* gene, we measured the mRNA expression levels of *DOCK7*, while circDOCK7 was overexpressed and knocked down in proliferated and differentiated chicken abdominal preadipocytes, respectively. A qRT-PCR analysis showed no significant change in the mRNA expression level of *DOCK7* gene in the proliferative and differentiated chicken abdominal preadipocytes upon circDOCK7 overexpression (Additional file 2: Fig. S4C) or interference (Additional file 2: Fig. S4D). This indicates that circDOCK7 could not regulate parental gene expression during adipogenesis in chickens.

**Fig. 4:**
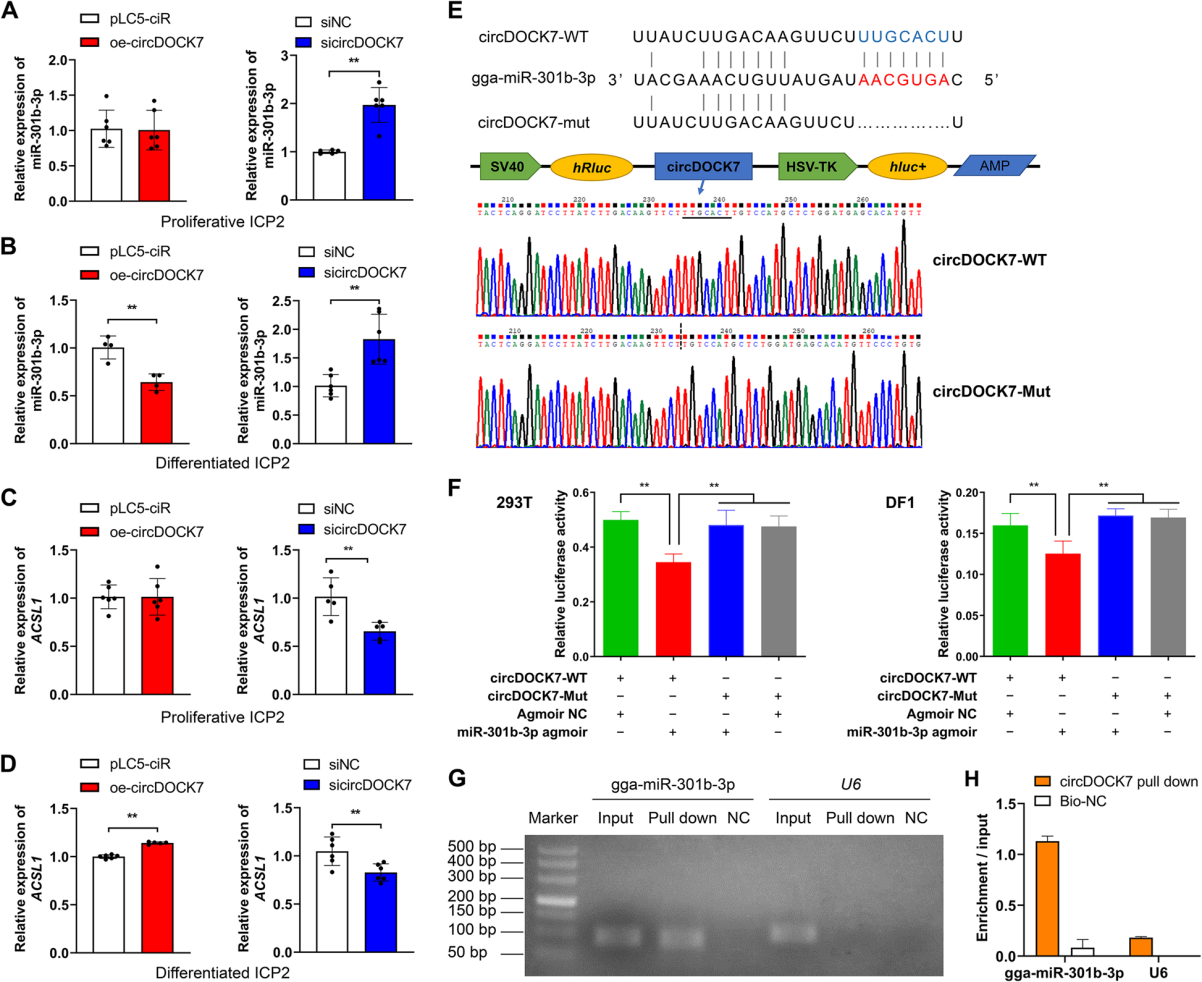
circDOCK7 functions as a gga-miR-301b-3p sponge. **A** and **B** Relative expression of gga-miR-301b-3p in proliferative and differentiated chicken abdominal preadipocytes upon circDOCK7 overexpression and knockdown. **C** and **D** Relative expression of the *ACSL1* gene in proliferative and differentiated chicken abdominal preadipocytes upon circDOCK7 overexpression and knockdown. **E** Construction of dual-luciferase reporter vectors for the validation of gga-miR-301b-3p binding to circDOCK7. WT: wild-type vector; Mut: mutant vector; *hRluc*: *Renilla* luciferase; *hluc* + : firefly luciferase. **F** Validation of the interaction between gga-miR-301b-3p and circDOCK7 via a dual-luciferase reporter assay in 293 T cells and DF1 cells. **G** Detection of the combination of circDOCK7 and gga-miR-301b-3p by an RNA pull-down assay. **H** qRT-PCR analysis of gga-miR-301b-3p expression pulled down by the circDOCK7 probe. ^*^P < 0.05, ^**^P < 0.01

Based on the expression profiles of circDOCK7, potential miRNAs absorbed by circDOCK7 and their potential target mRNAs, the constructed ceRNA regulatory network showed that circDOCK7 might interact with five miRNAs: gga-miR-1354, gga-miR-143-3p, gga-miR-301b-3p, novel_miR_105, and novel_miR_273, wherein gga-miR-301b-3p might target the *ACSL1* gene, an indispensable regulator at the critical juncture of de novo lipid synthesis that is involved in several lipid-related signaling pathways, including fatty acid metabolism, fatty acid biosynthesis, fatty acid degradation, PPAR signaling pathway, and adipocytokine signaling pathway (Additional file 2: Fig. S5A). Furthermore, circDOCK7 expression was significantly positively correlated (Pearson correlation coefficient = 0.8007, *P* = 0.0003) with *ACSL1* expression (Additional file 2: Fig. S5B and C). The MFEs of the RNA duplexes formed by circDOCK7 and gga-miR-301b-3p as well as gga-miR-301b-3p and *ACSL1* gene were −22.9 and −22.7 kcal/mol, respectively, indicating the high strength of these hybridizations (Additional file 2: Fig. S5D and E). Additionally, in proliferative chicken abdominal preadipocytes, the expression level of gga-miR-301b-3p was remarkably increased by circDOCK7 interference, although there was no significant change with circDOCK7 overexpression (Fig. 4A). In differentiated chicken abdominal preadipocytes induced by sodium oleate, the overexpression of circDOCK7 significantly inhibited gga-miR-301b-3p expression compared to the pLC5-ciR control group, whereas the knockdown of circDOCK7 significantly enhanced gga-miR-301b-3p expression compared to the siNC control group (Fig. 4B). In contrast, the *ACSL1* mRNA abundance was significantly decreased in both sicircDOCK7-treated proliferative and differentiated ICP2 cells compared to the siNC group, and was significantly increased in differentiated ICP2 cells transfected with circDOCK7 overexpression vector compared to the control group (Fig. 4C and D). This inspired us to explore the circDOCK7-mediated ceRNA regulation during chicken adipogenesis.

To confirm the interaction between circDOCK7 and gga-miR-301b-3p, we first predicted one gga-miR-301b-3p-binding site in the circDOCK7 sequences and constructed wild-type and mutant plasmids that contained and lacked this binding site, respectively (Fig. 4E). As expected, a dual-luciferase reporter assay revealed that co-transfection of miR-301b-3p agomir and wild-type circDOCK7 significantly reduced the relative luciferase activity in both 293 T and DF1 cells; however, the co-transfection of miR-301b-3p agomir and mutant circDOCK7 did not affect the relative luciferase activity (Fig. 4F). This demonstrated direct binding between circDOCK7 and gga-miR-301b-3p based on their complementary sequences. To further determine the direct interaction of circDOCK7 and gga-miR-301b-3p in chicken abdominal preadipocytes, we conducted a pull-down experiment in chicken abdominal preadipocytes and confirmed the enrichment of gga-miR-301b-3p in the circDOCK7 probe-treated group, but not in the Bio-NC group (Fig. 4G). Moreover, gga-miR-301b-3p expression was remarkably increased in the circDOCK7 probe-treated group compared to the Bio-NC group, as determined by qRT-PCR (Fig. 4H). These results suggested that circDOCK7 directly binds to gga-miR-301b-3p in chicken abdominal preadipocytes.

### gga-miR-301b-3p inhibits the proliferation of chicken abdominal preadipocytes

**Fig. 5:**
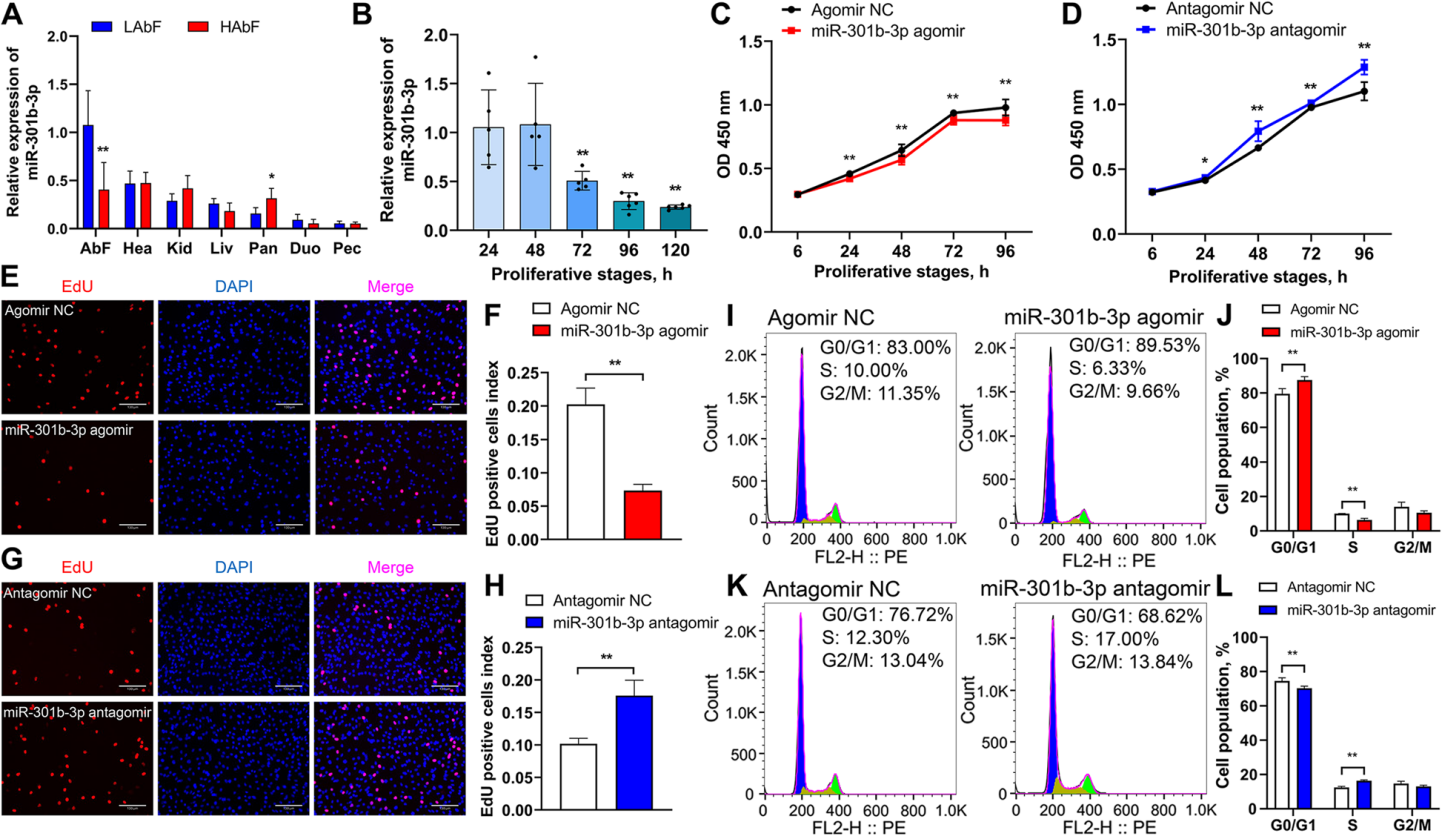
Effects of gga-miR-301b-3p on the proliferation of chicken abdominal preadipocytes. **A** Tissue expression pattern of gga-miR-301b-3p in chickens with high and low abdominal fat percentage. **B** Expression pattern of gga-miR-301b-3p during the proliferation of chicken abdominal preadipocytes. **C** CCK8 assay of chicken abdominal preadipocytes transfected with miR-301b-3p agomir and miR-301b-3p agomir NC. **D** CCK8 assay of chicken abdominal preadipocytes transfected with miR-301b-3p antagomir and miR-301b-3p antagomir NC. **E** Proliferation of chicken abdominal preadipocytes determined by EdU after 48 h of transfection with miR-301b-3p agomir and miR-301b-3p agomir NC. **F** Histogram showing the proportion of gga-miR-301b-3p-overexpressing EdU-positive cells using ImageJ. **G** Proliferation of chicken abdominal preadipocytes determined by EdU after 48 h of transfection with miR-301b-3p antagomir and miR-301b-3p antagomir NC. **H** Histogram showing the proportion of gga-miR-301b-3p-knockdown EdU-positive cells using ImageJ. **I** and **J** Flow-cytometric cell cycle analysis of chicken abdominal preadipocytes after 48 h of transfection with miR-301b-3p agomir and miR-301b-3p agomir NC. **K** and **L** Flow-cytometric cell cycle analysis of chicken abdominal preadipocytes after 48 h of transfection with miR-301b-3p antagomir and miR-301b-3p antagomir NC. ^*^P < 0.05, ^**^P < 0.01

To determine whether gga-miR-301b-3p functions in chicken adipogenesis, we first monitored the tissue expression profiles of HAbF and LAbF broilers. A qRT-PCR assay showed that gga-miR-301b-3p expression was the most abundant in abdominal fat among the seven detected tissues and was significantly downregulated in the abdominal fat of HAbF broilers than that of LAbF broilers (Fig. 5A). We then detected gga-miR-301b-3p expression in chicken abdominal preadipocytes at different stages of proliferation and conducted gain- and loss-of-function experiments by transfecting miR-301b-3p agomir and miR-301b-3p antagomir into ICP2 cells, respectively. The results showed that gga-miR-301b-3p expression was gradually downregulated during chicken abdominal preadipocyte proliferation in a time-dependent manner (Fig. 5B). A CCK8 assay showed that gga-miR-301b-3p overexpression significantly decreased the number of living ICP2 cells at 24, 48, 72, and 96 h post-transfection, whereas gga-miR-301b-3p knockdown significantly increased the number of living ICP2 cells (Fig. 5C and D). Similarly, EdU staining revealed that remarkably fewer EdU-positive cells were accompanied by gga-miR-301b-3p overexpression (Fig. 5E and F), contrary to those triggered by gga-miR-301b-3p interference (Fig. 5G and H). Additionally, a cell cycle analysis showed that the proportion of ICP2 cells was significantly increased in the G0/G1 phase and significantly decreased in the S phase upon gga-miR-301b-3p overexpression (Fig. 5I and J); gga-miR-301b-3p interference exhibited the opposite effects (Fig. 5K and L). These results indicated that gga-miR-301b-3p inhibited the proliferation of chicken abdominal preadipocytes by blocking their progression from the G0/G1 phase to the S phase.

### gga-miR-301b-3p suppresses the adipogenic differentiation of chicken abdominal preadipocytes

**Fig. 6:**
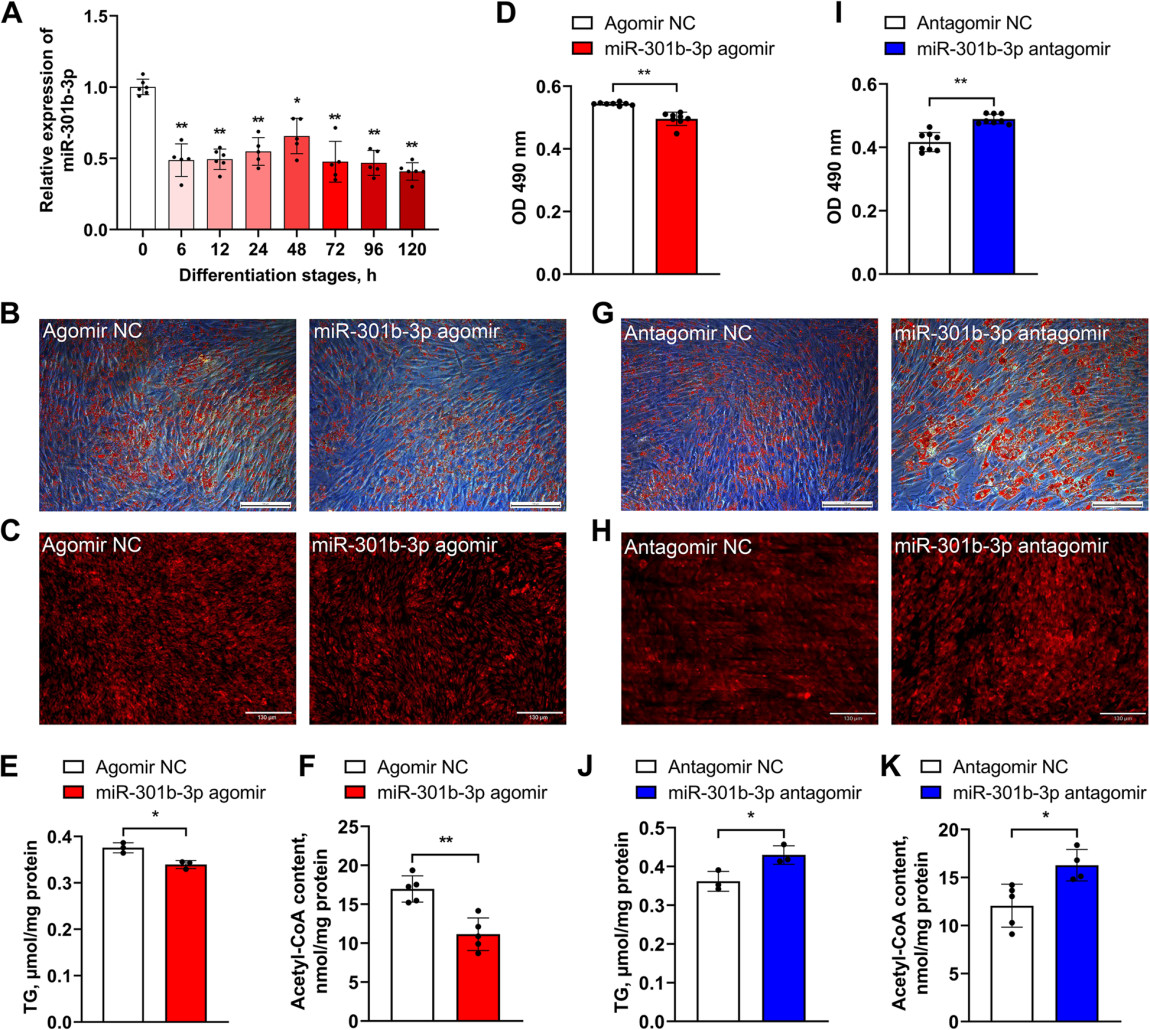
Effects of gga-miR-301b-3p on the adipogenic differentiation of chicken abdominal preadipocytes. **A** Expression pattern of gga-miR-301b-3p during the adipogenic differentiation of chicken abdominal preadipocytes. **B**, **C** Representative images of Oil Red O staining and Nile red fluorescent staining of gga-miR-301b-3p-overexpressing differentiated chicken abdominal adipocytes. **D** Detection of lipid droplet content of gga-miR-301b-3p-overexpressing differentiated chicken abdominal adipocytes. **E** and **F** Intracellular triglyceride and acetyl-CoA levels of gga-miR-301b-3p-overexpressing differentiated chicken abdominal adipocytes. **G** and **H** Representative images of Oil Red O staining and Nile red fluorescent staining of gga-miR-301b-3p-knockdown differentiated chicken abdominal adipocytes. **I** Detection of the lipid droplet content of gga-miR-301b-3p-knockdown differentiated chicken abdominal adipocytes. **J **and **K** Intracellular triglyceride and acetyl-CoA levels of gga-miR-301b-3p-knockdown differentiated chicken abdominal adipocytes. ^*^P < 0.05, ^**^P < 0.01

To further assess the functions of gga-miR-301b-3p in chicken adipogenesis, we monitored its expression pattern during the adipogenic differentiation of chicken abdominal preadipocytes and the changes in intracellular acetyl-CoA, triglyceride, and lipid droplet accumulation in differentiated ICP2 cells with gga-miR-301b-3p overexpression and knockdown, respectively. Here, gga-miR-301b-3p was significantly downregulated in differentiated than in undifferentiated chicken abdominal preadipocytes (Fig. 6A). A significant decrease in intracellular lipid droplet content was detected in ICP2 cells transfected with gga-miR-301b-3p agomir for 48 h, as determined by Oil Red O and Nile red fluorescent staining assays (Fig. 6B–D). Additionally, intracellular triglyceride and acetyl-CoA levels were significantly lower in the gga-miR-301b-3p overexpression group than in the control group (Fig. 6E, F). In contrast, intracellular lipid droplet, triglyceride, and acetyl-CoA levels were significantly increased in the gga-miR-301b-3p knockdown group than in the control group (Fig. 6G–K). These results indicate that gga-miR-301b-3p suppresses the adipogenic differentiation of abdominal preadipocytes in chickens.

### *ACSL1* is a direct target of gga-miR-301b-3p

**Fig. 7:**
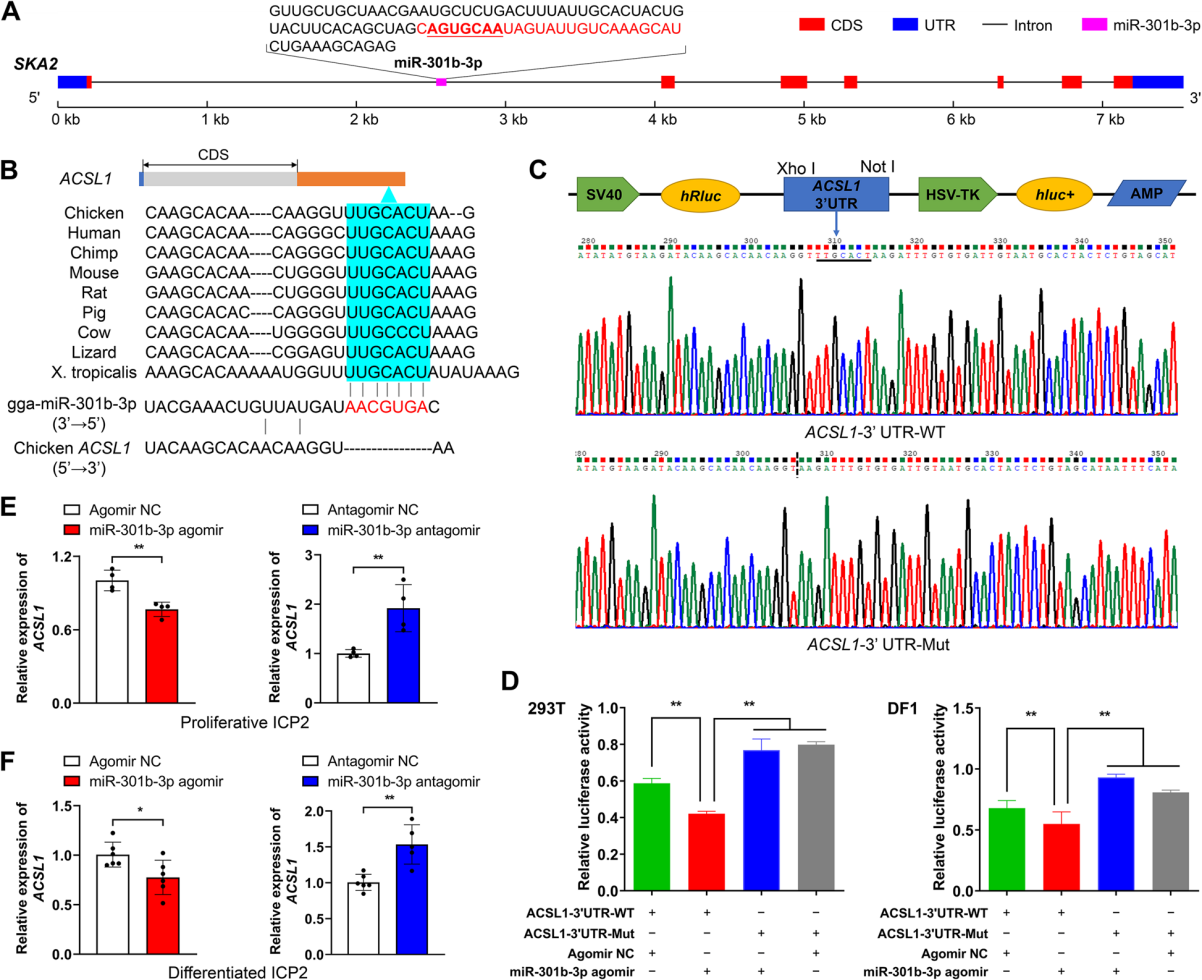
Validation of the *ACSL1* gene as a direct target of gga-miR-301b-3p. **A** Schematic diagram of the genomic location of gga-miR-301b-3p. The sequences encoding the precursor gga-miR-301b-3p are shown, with the mature gga-miR-301b-3p sequences highlighted in red. **B** Complementary sequences of gga-miR-301b-3p and the 3′ UTR of the *ACSL1* gene of different species. The seed region of gga-miR-301b-3p is highlighted in red; potential gga-miR-301b-3p-binding sites in the 3′ UTR of the *ACSL1* gene are shown in blue. **C** Construction of dual-luciferase reporter vectors for the validation of gga-miR-301b-3p targeting the *ACSL1* gene. WT: wild-type vector; mut: mutant vector; *hRluc*: *Renilla* luciferase; *hluc* + : firefly luciferase. **D** Validation of the interaction between gga-miR-301b-3p and the 3′ UTR of the *ACSL1* gene by a dual-luciferase reporter assay in 293 T cells and DF1 cells. **E** Relative *ACSL1* mRNA expression in gga-miR-301b-3p-overexpressing and -knockdown proliferative chicken abdominal preadipocytes. The relative gene expression levels are shown as fold changes compared with that in the agomir NC and antagomir NC groups, respectively. The same below. **F** Relative *ACSL1* mRNA expression in gga-miR-301b-3p-overexpressing and -knockdown differentiated chicken abdominal preadipocytes. ^*^P < 0.05, ^**^P < 0.01

The miRNA gga-miR-301b-3p was derived from intron 1 of the spindle and kinetochore-associated complex subunit 2 pseudogene 1 (*SKA2*) gene in the chicken genome (Fig. 7A). Our results suggested that the seed region gga-miR-301b-3p could bind to the 3′ untranslated region (UTR) of the *ACSL1* gene, which is conserved among species, including human, chimp, mouse, rat, pig, cow, lizard and western clawed from (*Xenopus tropicalis*) (Fig. 7B). To confirm the interaction between gga-miR-301b-3p and *ACSL1*, we constructed wild-type and mutant dual-luciferase reporter plasmids that contained and lacked the binding sites of the gga-miR-301b-3p seed region in the *ACSL1* gene 3′-UTR, named ACSL1-3′UTR-WT and ACSL1-3′UTR-Mut, respectively (Fig. 7C). The dual-luciferase reporter plasmids were co-transfected with miR-301b-3p agomir or agomir NC into 293 T and DF1 cells. As expected, a dual-luciferase reporter assay revealed that gga-miR-301b-3p significantly decreased the relative luciferase activity of ACSL1-3′UTR-WT but did not affect that of ACSL1-3′UTR-Mut in either 293 T cells or DF1 cells (Fig. 7D). Moreover, the *ACSL1* mRNA levels were significantly suppressed upon miR-301b-3p overexpression and significantly enhanced upon miR-301b-3p knockdown in both proliferative and differentiated chicken abdominal preadipocytes (Fig. 7E and F). These results indicated that gga-miR-301b-3p directly targeted the *ACSL1* gene and inhibited its post-transcriptional expression.

### Subcellular localization and expression profiles of chicken ACSL1 protein

**Fig. 8:**
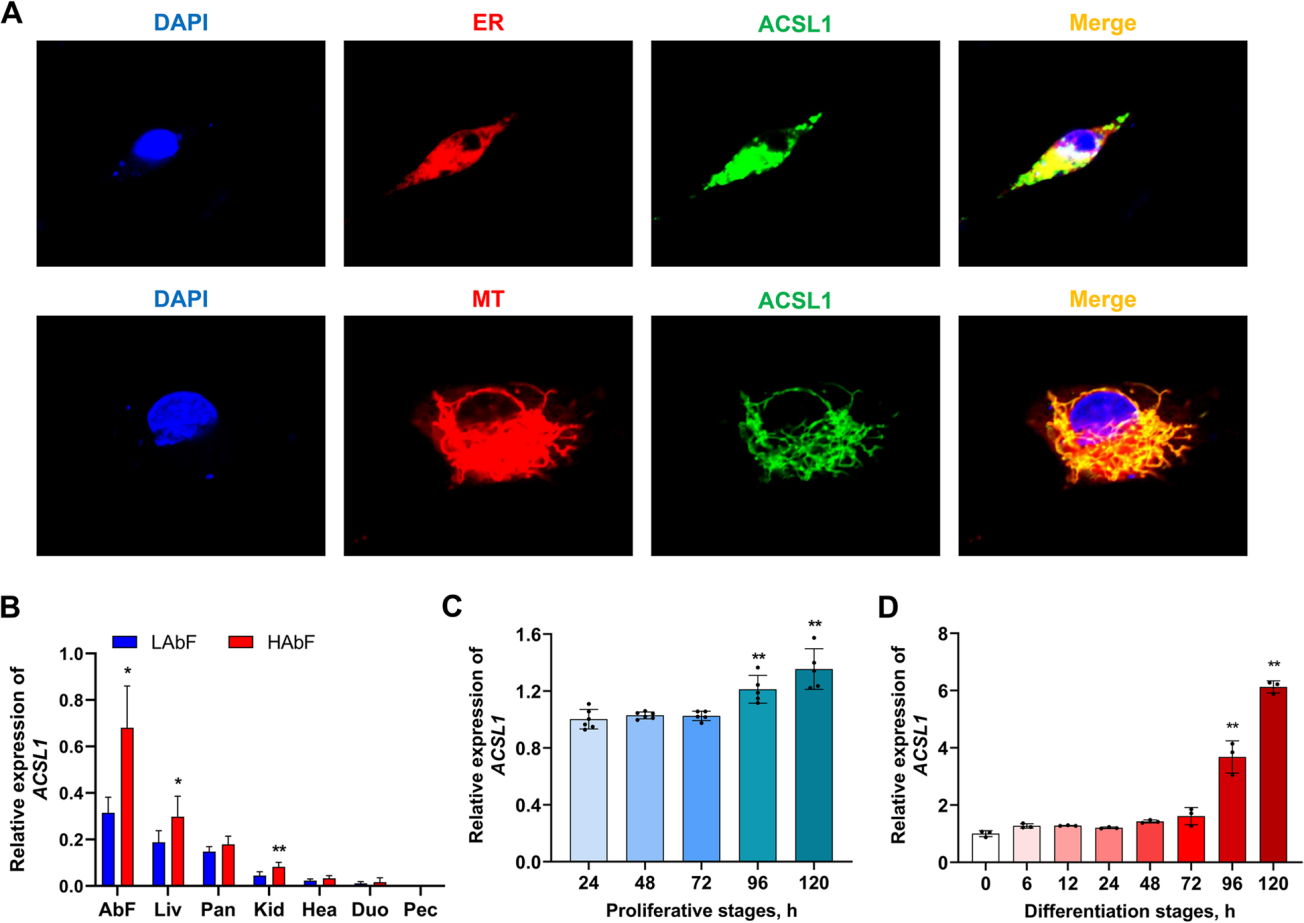
Subcellular localization and expression profiles of chicken ACSL1 protein. **A** Subcellular localization of ACSL1 in chicken abdominal preadipocytes. MT represents mitochondrion. ER represents endoplasmic reticulum. Cell nuclei were stained with DAPI. **B** Tissue expression pattern of the *ACSL1* gene in chickens with high and low abdominal fat percentage. **C** and **D** Expression pattern of *ACSL1* during the proliferation and adipogenic differentiation of chicken abdominal preadipocytes. ^*^P < 0.05, ^**^P < 0.01

Most proteins can exert their functions in multiple compartments, which is determined by their subcellular localization. To determine the subcellular localization of the chicken ACSL1 protein, we first predicted it using UniProt and found that chicken ACSL1 was likely located in the endoplasmic reticulum, mitochondria, and membranes (Additional file 2: Fig. S6). Subsequently, our fluorescence staining assays showed that the chicken ACSL1 protein was indeed distributed in the endoplasmic reticulum and mitochondria of chicken abdominal preadipocytes (Fig. 8A). The tissue expression profiles of the *ACSL1* gene in HAbF and LAbF broilers showed that *ACSL1* exhibited the highest mRNA expression in abdominal fat and was significantly upregulated in the abdominal fat tissues of the HAbF group compared to the LAbF group (Fig. 8B). Moreover, *ACSL1* showed a gradual increase in mRNA expression during the proliferation and adipogenic differentiation of chicken abdominal preadipocytes (Fig. 8C and D). These results suggest that *ACSL1* plays a crucial role in abdominal adipogenesis in chickens.

### *ACSL1* gene facilitates proliferation and glucose uptake in chicken abdominal preadipocytes

**Fig. 9:**
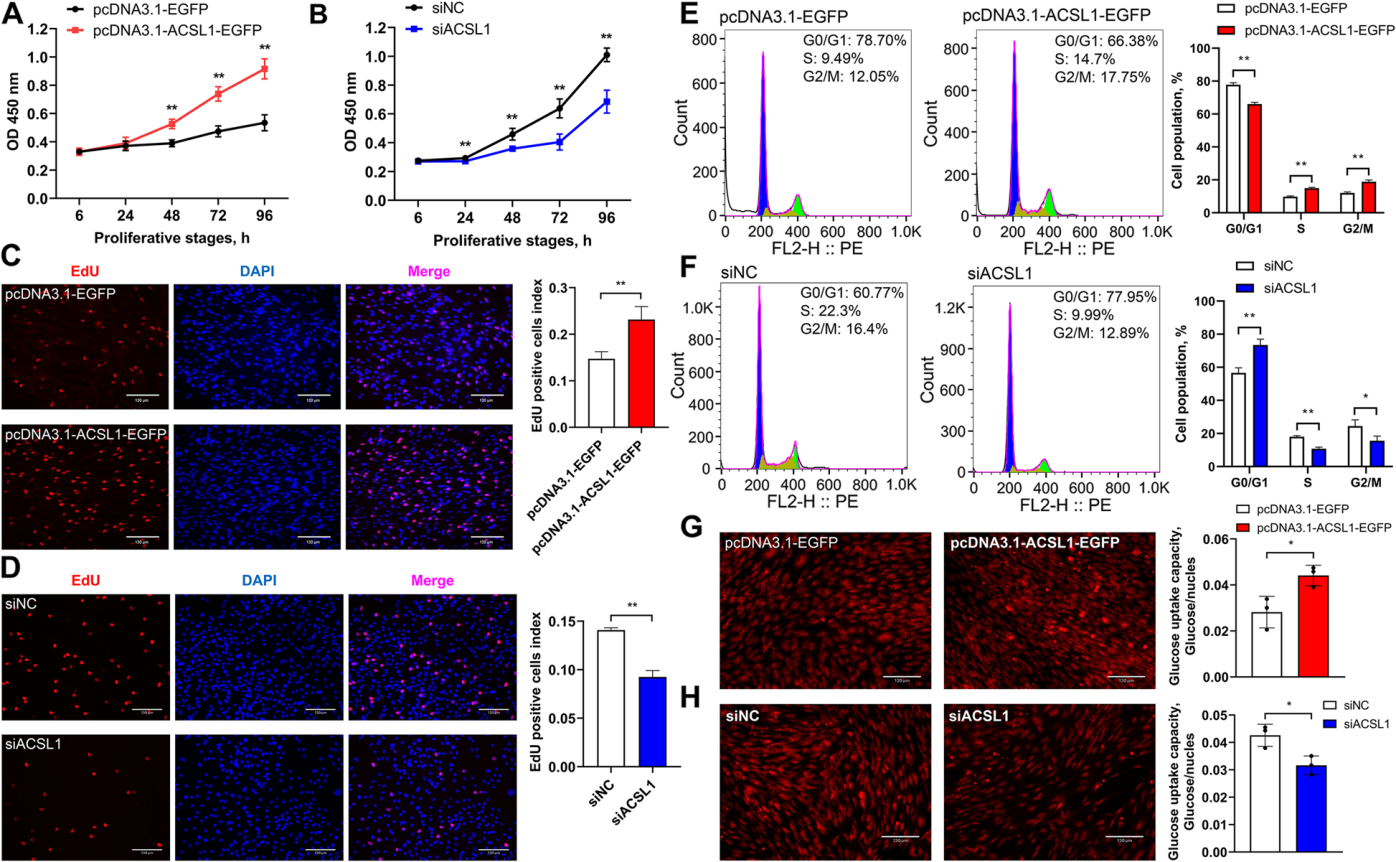
Effects of *ACSL1* gene expression on the proliferation of chicken abdominal preadipocytes. **A** and **B** CCK8 assay of *ACSL1*-overexpressing and -knockdown chicken abdominal preadipocytes. **C **and **D** Proliferation of *ACSL1*-overexpressing and -knockdown chicken abdominal preadipocytes determined by EdU assay. **E** and **F** Flow-cytometric cell cycle analysis of *ACSL1*-overexpressing and -knockdown chicken abdominal preadipocytes. **G** and **H** Glucose uptake capacity of *ACSL1*-overexpressing and -knockdown chicken abdominal preadipocytes. ^*^P < 0.05, ^**^P < 0.01

To explore the effects of the *ACSL1* gene on the proliferation of chicken abdominal preadipocytes, we performed gain- and loss-of-function experiments by transfecting the overexpression vector and siRNA into ICP2 cells. The number of living cells was significantly increased upon *ACSL1* overexpression but was significantly decreased upon *ACSL1* silencing, as determined by the CCK8 assay (Fig. 9A and B). Accordingly, EdU staining showed that the proportion of EdU-positive chicken abdominal preadipocytes was markedly increased by *ACSL1* overexpression and markedly decreased by *ACSL1* silencing (Fig. 9C and D). Moreover, cell cycle analysis demonstrated that *ACSL1* overexpression promoted normal cell cycle progression characterized by significantly decreased G0/G1-phase and significantly increased S- and G2/M-phase cell populations (Fig. 9E). In contrast, *ACSL1* silencing produced the opposite effect and inhibited the normal cell cycle progression (Fig. 9F). However, the intracellular glucose uptake capacity of chicken abdominal preadipocytes was significantly increased by *ACSL1* overexpression and significantly decreased by *ACSL1* interference (Fig. 9G and H). These results indicated that the *ACSL1* gene could inhibit the proliferation of chicken abdominal preadipocytes by accelerating the cell cycle transition from G0/G1 to S phase and S to G2/M phase as well as the intracellular glucose uptake capacity of chicken abdominal preadipocytes.

### *ACSL1* gene facilitates the adipogenic differentiation of chicken abdominal preadipocytes

**Fig. 10:**
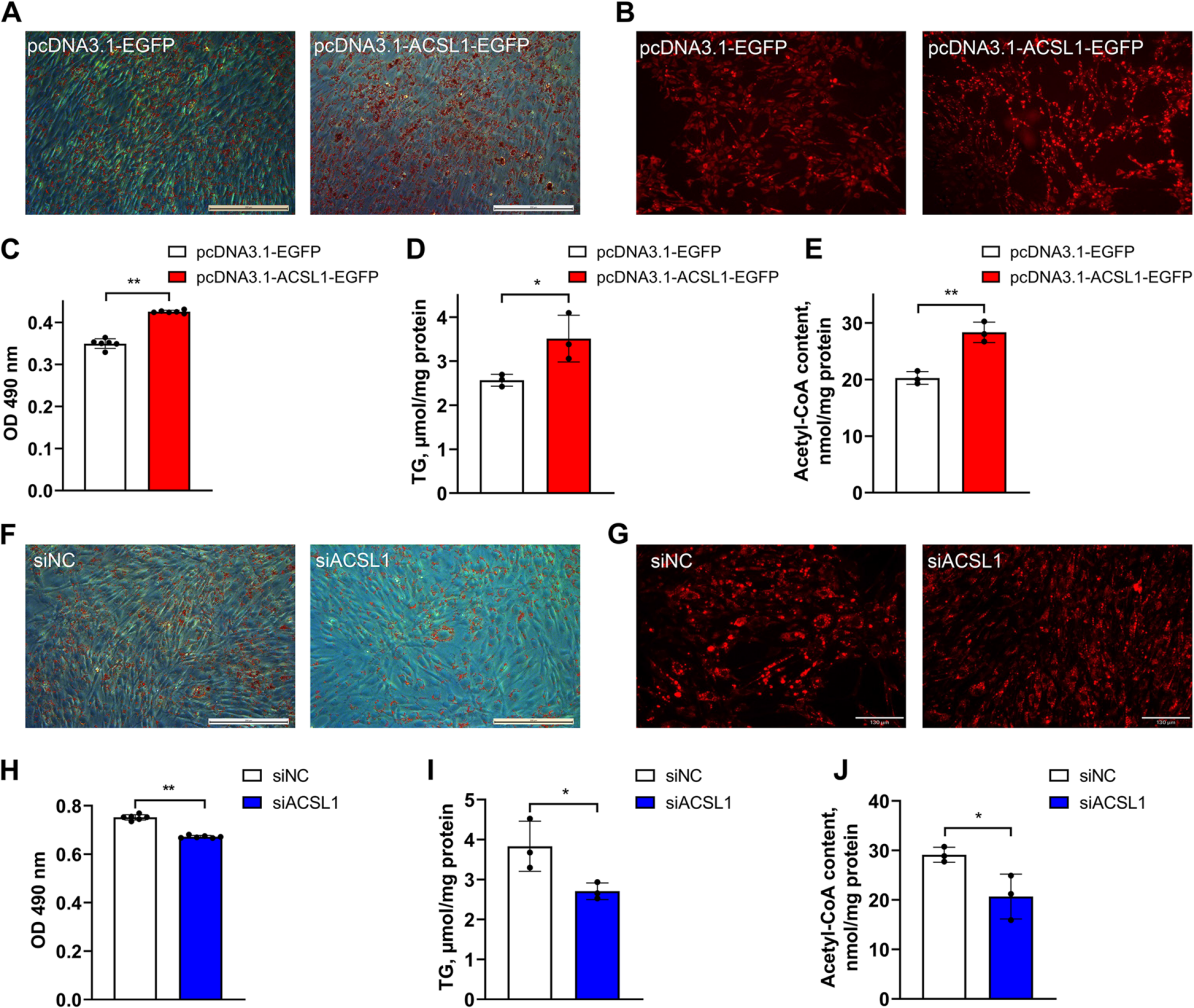
Effects of *ACSL1* gene expression on the adipogenic differentiation of chicken abdominal preadipocytes. **A** and **B** Representative images of Oil Red O staining and Nile red fluorescent staining of *ACSL1*-overexpressing differentiated chicken abdominal adipocytes. **C** Detection of the lipid droplet content of *ACSL1*-overexpressing differentiated chicken abdominal adipocytes. **D** and **E** Intracellular triglyceride and acetyl-CoA levels of *ACSL1*-overexpressing differentiated chicken abdominal adipocytes. **F** and **G** Representative images of Oil Red O staining and Nile red fluorescent staining of *ACSL1*-knockdown differentiated chicken abdominal adipocytes. **H** Detection of the lipid droplet content of *ACSL1*-knockdown differentiated chicken abdominal adipocytes. **I** and **J** Intracellular triglyceride and acetyl-CoA levels of *ACSL1*-knockdown differentiated chicken abdominal adipocytes. ^*^P < 0.05, ^**^P < 0.01

To confirm whether *ACSL1* regulates the adipogenic differentiation of chicken abdominal preadipocytes, we detected intracellular lipid droplet accumulation as well as intracellular triglyceride and acetyl-CoA levels via gain- and loss-of-function experiments in differentiated ICP2 cells. Excessive *ACSL1* expression induced a significant increase in intracellular lipid droplet accumulation, as determined by Oil Red O and Nile red fluorescent staining assays (Fig. 10A–C). Moreover, intracellular triglyceride and acetyl-CoA levels were significantly increased upon *ACSL1* stimulation (Fig. 10D and E). Accordingly, intracellular lipid droplet accumulation and triglyceride and acetyl-CoA levels were significantly lower in the siACSL1 knockdown group than in the siNC control group (Fig. 10F–J). These results indicate that *ACSL1* facilitates the adipogenic differentiation of abdominal preadipocytes in chickens.

### CircDOCK7 augments adipogenesis via acting as a ceRNA of gga-miR-301b-3p to activate the *ACSL1* gene

**Fig. 11:**
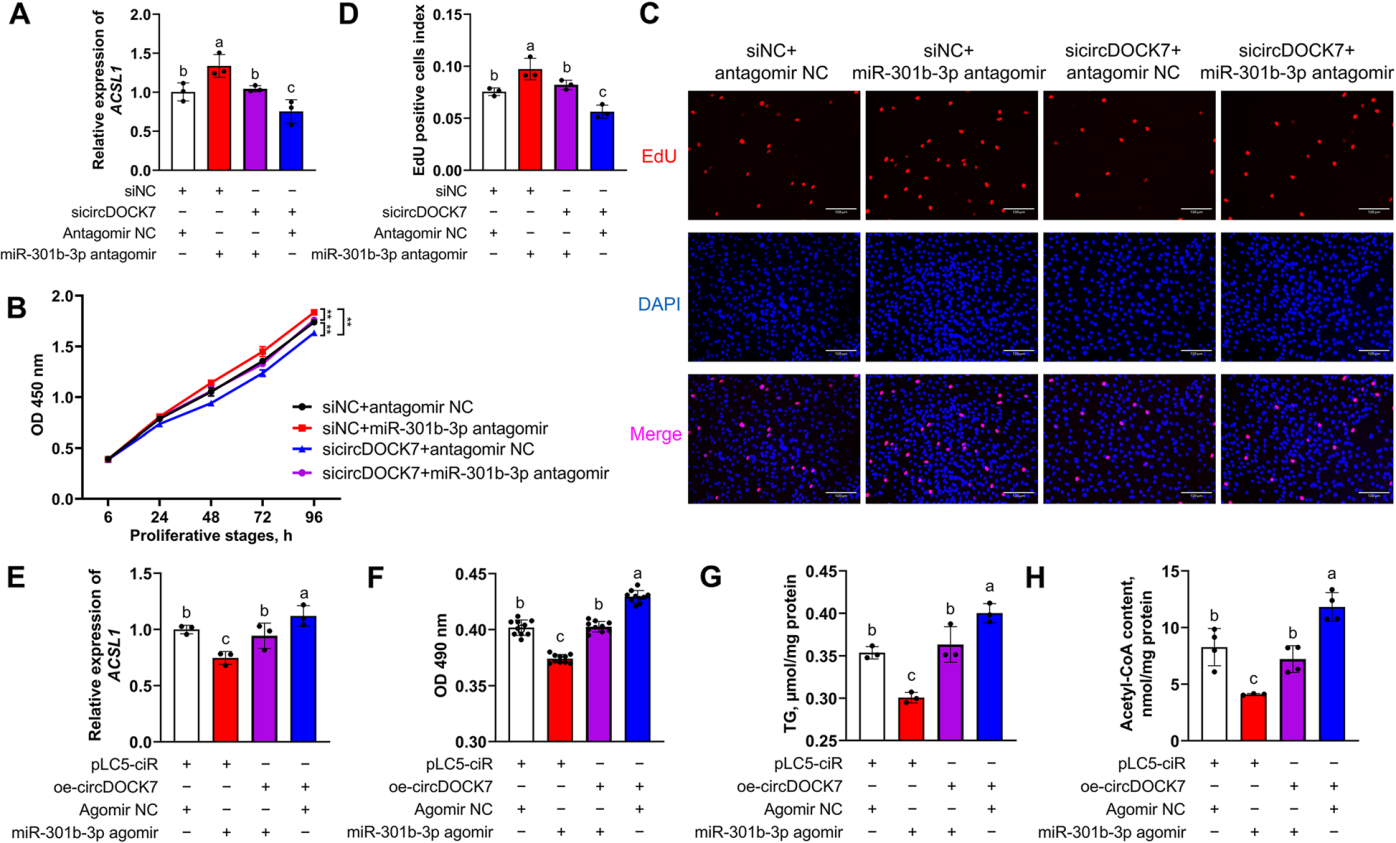
circDOCK7 augments adipogenesis in a gga-miR-301b-3p-dependent manner. **A** Relative expression of the *ACSL1* gene in proliferative chicken abdominal preadipocytes co-transfected with sicircDOCK7 and miR-301b-3p antagomir. The relative gene expression levels are shown as fold changes compared with that in the siNC + antagomir NC group. **B**–**D** CCK8 and EdU staining assays in proliferative chicken abdominal preadipocytes co-transfected with sicircDOCK7 and miR-301b-3p antagomir. **E** Relative expression of the *ACSL1* gene in differentiated chicken abdominal preadipocytes co-transfected with circDOCK7 overexpression vector and miR-301b-3p agomir. The relative gene expression levels are shown as fold changes compared with that in the pLC5-ciR + agomir NC group. **F–H** Intracellular lipid droplet accumulation and triglyceride and acetyl-CoA levels in differentiated chicken abdominal adipocytes co-transfected with circDOCK7 overexpression vector and miR-301b-3p agomir. ^*^P < 0.05, ^**^P < 0.01. ^a–c^Means with different lowercase letters indicate significant difference (*P* < 0.05)

Based on the interaction of gga-miR-301b-3p with circDOCK7 and *ACSL1*, we suggested that circDOCK7 might affect the abdominal adipogenesis via severing as a “miRNA sponge” of gga-miR-301b-3p to regulate the *ACSL1* gene in chickens. To verify this, ICP2 cells were co-transfected with siNC + antagomir NC, siNC + miR-301b-3p antagomir, sicircDOCK7 + antagomir NC, and sicircDOCK7 + miR-301b-3p antagomir. At 48 h post-transfection, we determined *ACSL1* mRNA expression and found that the miR-301b-3p antagomir-induced increase in *ACSL1* mRNA expression was rescued by circDOCK7 knockdown, and the inhibitory role of circDOCK7 knockdown on *ACSL1* mRNA expression was alleviated by miR-301b-3p antagomir (Fig. 11A), indicating that circDOCK7 could act as a ceRNA of gga-miR-301b-3p to post-transcriptionally regulate the downstream target *ACSL1*. Subsequently, we analyzed the cell proliferation of chicken abdominal preadipocytes and found that it was significantly suppressed by circDOCK7 knockdown, as evidenced by the CCK8 and EdU assays, which was abolished after supplemental treatment with gga-miR-301b-3p antagomir. Additionally, the gga-miR-301b-3p-silence-mediated promotion of the proliferation of chicken abdominal preadipocytes was abrogated following supplemental circDOCK7 knockdown (Fig. 11B–D).

ICP2 cells were co-transfected with pLC5-ciR + miR-301b-3p agomir NC, pLC5-ciR + miR-301b-3p agomir, oe-circDOCK7 + miR-301b-3p agomir NC, and oe-circDOCK7 + miR-301b-3p agomir. At 48 h post-transfection, we detected *ACSL1* mRNA expression and found that circDOCK7 overexpression promoted *ACSL1* mRNA expression in differentiated chicken abdominal preadipocytes, whereas this effect was abolished by miR-301b-3p agomir. In addition, the suppression of *ACSL1* mRNA expression caused by the miR-301b-3p agomir was rescued by circDOCK7 overexpression (Fig. 11E). Meanwhile, circDOCK7 overexpression reversed miR-301b-3p overexpression-mediated inhibition of intracellular lipid droplet accumulation as well as reduction in intracellular triglyceride and acetyl-CoA levels, and vice versa (Fig. 11F–H). Taken together, these results illustrate that the gga-miR-301b-3p/*ACSL1* axis is required for the function of circDOCK7, and circDOCK7 could augment chicken abdominal adipogenesis in vitro by acting as a ceRNA, competitively adsorbing gga-miR-301b-3p to sequester it away from the *ACSL1* gene, thus post-transcriptionally activating *ACSL1* gene expression.

## Discussion

Abnormal or excessive abdominal fat deposition is of tremendous concern owing to the worldwide prevalence of obesity and obesity-associated escalating health burdens, such as type 2 diabetes, dyslipidemia, hypertension, cardiovascular disease, and even cancers. In commercial broilers, abdominal fat is an imperative carcass trait, and its excessive accumulation is often discarded as a waste of feed energy, which increases their tendency toward physiological disorders and presents an obstacle to profitable farming. Despite substantial research efforts aimed at characterizing the genetic regulation of obesity and adipogenesis, the molecular mechanisms underlying abdominal fat deposition remain largely unknown in mammals and economically important agricultural avian species, especially chickens. CircRNAs are ubiquitous across eukaryotes and have been increasingly implicated as master regulators of mammalian adipogenesis [41, 42]. A number of circRNAs have been reported to share high evolutionary conservation among closely related species, even distantly related species [43]. Chicken (*Gallus gallus*) has been used as a good biomedical model organism to study the basic mechanisms of adipogenesis, obesity, and obesity-related diseases [44] and is suitable for investigating the circRNA-mediated biological functions and regulatory mechanisms underlying adipogenesis, which can produce useful comparative information for human obesity studies and also provide a promising biomarker associated with abdominal fat deposition for lean-line broiler breeding.

Abdominal fat deposition depends on the proliferation of preadipocytes and their maturation into adipocytes, during which an increase in lipid (mainly triglycerides) synthesis in adipocytes is a hallmark of adipogenic differentiation compared to preadipocytes. Our previous study based on circRNA sequencing found that circRNAs were abundant and dynamically expressed during abdominal preadipocyte differentiation in chickens [27]. Among these, an exonic circRNA, circDOCK7, exhibited a gradual increase in expression in abdominal adipocytes at different adipogenic differentiation stages, suggesting that circDOCK7 may play a crucial role in the positive regulation of adipogenesis in chickens. Backsplicing junction verification, RNase R treatment, and actinomycin D treatment confirmed that circDOCK7 is more stable than its linear counterpart, in agreement with the circular characteristics of circRNAs owing to their closed-loop structure, RNA exonuclease resistance, and longer half-lives [6, 45]. Our expression pattern analysis also demonstrated that circDOCK7 exhibited an overall increase in expression during the proliferation and differentiation of chicken abdominal preadipocytes. Moreover, circDOCK7 was most abundantly expressed and significantly upregulated in the abdominal fat tissues of chickens with a high abdominal fat percentage compared to that of chickens with a low abdominal fat percentage. Consistent with this, our in vitro gain- and loss-of-function experiments confirmed that circDOCK7 not only enhanced the proliferation of chicken abdominal preadipocytes, but also increased adipocyte lipid droplet accumulation and triglyceride, and acetyl-CoA content, in parallel with the mRNA abundance of *PPARγ* and *CEBPα*, two crucial determinants necessary and sufficient for adipocyte differentiation [46]. These results indicate that circDOCK7 serves as an accelerator of adipogenesis, resulting in the proliferation and differentiation of abdominal preadipocytes in chickens.

Once produced, circRNAs are either released into the cytoplasm or retained inside the nucleus, and exonic circRNAs predominantly localize to the cytoplasm [47]. Consistent with this, circDOCK7 is an exonic circRNA harboring three exons derived from *DOCK7* and is mainly located in the cytoplasm of chicken abdominal preadipocytes. It has been proposed that the back-splicing of exons could compete against linear mRNA splicing during transcription; therefore, exonic circRNA biogenesis can completely alter the expression of their linear gene product [48]. Recent studies have also demonstrated the activity of some exonic circRNAs as protein recruiters that recruit specific proteins to certain loci or subcellular compartments, eventually influencing gene expression [49]. A case in point is that a novel circular RNA consisting of exons 4–2-3 of the friend leukemia virus integration 1 (*FLI1*) gene, referred to as FECR1, could recruit ten-eleven translocation methylcytosine dioxygenase 1 (TET1), a demethylase that is actively involved in DNA demethylation, to the promoter region of its host *FLI1* gene, leading to DNA hypomethylation of CpG islands and active *cis* transcription of the *FLI1* gene, thus regulating the metastasis of breast cancer cells [50]. CircDOCK7 is derived from the backsplicing of exons 19–22 of its parental *DOCK7* gene. *DOCK7* gene encoded a guanine nucleotide exchange factor for the Rho family of GTPases and has been used for type 2 diabetic obese model mice [51]. It was reported that *DOCK7* gene polymorphism was dramatically associated with serum lipid levels including triglyceride, total cholesterol, low-density lipoprotein cholesterol, apolipoprotein B in humans [52, 53]. In the present study, the *DOCK7* gene exhibited no significant change during adipogenic differentiation of chicken abdominal preadipocytes according to our published RNA sequencing data (accession number: PRJNA732104) [27]. There is no significant expression correlation between the expression levels of circDOCK7 and *DOCK7* gene during adipogenic differentiation of chicken abdominal preadipocytes. Importantly, the *DOCK7* expression levels were not significantly changed upon circDOCK7 overexpression or knockdown in either proliferative or differentiated chicken abdominal preadipocytes. Taken together, these results suggest almost no potential for impairing circDOCK7 on chicken abdominal adipogenesis by transcriptional regulation of its parental *DOCK7* gene, at least via competition with linear splicing and protein recruitment.

Although circRNAs were originally believed to be untranslatable owing to their lack of essential elements containing a 5′ cap and 3’ poly(A) tail for canonical cap-dependent translation initiation, it has been reported that some exonic circRNAs are equipped with an ORF. Accumulating evidence has shown that circRNAs are associated with ribosomes, confirming the authenticity of circRNA-translated functional peptides or proteins in mammalian cells [49, 54]. The translation of circRNAs is driven by an IRES element, which is a cis-acting RNA sequence, or m^6^A RNA modification, which is a post-transcriptional methylation modification that involves the addition of a methyl group to the N6 position of RNA adenosine, both of which can recruit the internal entry of small ribosomal subunits in eukaryotic cells to initiate translation [55]. To explore whether circDOCK7 can be translated into peptides or proteins, we carried out a bioinformatics analysis of ORF, IRES elements, and m^6^A modification based on the circDOCK7 sequence. However, despite the presence of an IRES element and two m^6^A modification sites, no ORF was detected in the sequence of circDOCK7, leading us to speculate that circDOCK7 has almost no translational capacity.

In addition, extensive evidence has supported the ceRNA hypothesis that cytoplasmic exonic circRNAs function as miRNA sponges or decoys that competitively adsorb miRNAs to protect target mRNAs from miRNA-dependent degradation, thereby modulating post-transcriptional regulation in various biological processes. miRNAs constitute a subclass of 18–25-nucleotide endogenous single-stranded non-coding RNAs and have been implicated in the regulation of mammalian and avian adipogenesis, thereby gaining prominence as key oncogenic drivers [56, 57]. Some circRNAs have been reported to act as miRNA sponges that participate in mammalian adipogenesis, such as circSAMD4A [14], CDR1as [16], circFUT10 [58], sus_circPAPPA2 [19] and circPPARA [20]. CircRNAs were indeed expressed in abdominal fat tissues and adipocytes, and the ceRNA network comprising circRNAs, miRNAs, and mRNAs indicated that circRNAs may play regulatory roles in abdominal adipogenesis by interacting with one or more miRNAs to prevent them from executing their regulatory roles [24–27]. A sequence analysis revealed the presence of multiple predicted miRNA response elements in circDOCK7, which inspired us to explore the ceRNA regulatory mechanism of circDOCK7 underlying chicken abdominal fat deposition. Among these, gga-miR-301b-3p was validated as directly binding to circDOCK7 based on a pull-down assay and a dual-luciferase reporter assay. Moreover, circDOCK7 knockdown remarkably upregulated gga-miR-301b-3p expression in proliferative and differentiated chicken abdominal preadipocytes, confirming that circDOCK7 serves as a molecular sponge to adsorb gga-miR-301b-3p. The seed region of gga-miR-301b-3p is conserved among human, mouse, chimp, pig and cow, indicating a similar contribution to adipogenesis in mammals and chickens. In this study, gga-miR-301b-3p was significantly downregulated in abdominal fat tissues and showed an overall decreased expression level following the proliferation and adipogenic differentiation of chicken abdominal preadipocytes, which was strongly negatively correlated with circDOCK7, suggesting the involvement of gga-miR-301b-3p in abdominal adipogenesis in chickens. In this study, we demonstrated that chicken abdominal preadipocyte proliferation, adipocyte lipid droplet accumulation, and triglyceride and acetyl-CoA content were significantly decreased upon gga-miR-301b-3p stimulation but significantly increased upon gga-miR-301b-3p silencing, demonstrating that gga-miR-301b-3p served as an inhibitor of adipogenesis in chickens, in contrast to the role of circDOCK7. Strikingly, these inhibitory effects of gga-miR-301b-3p on the proliferation and adipogenic differentiation of chicken abdominal preadipocytes could be relieved by circDOCK7, and thus the enhanced effects of circDOCK7 could be abolished by miR-301b-3p. These results further suggested that circDOCK7 could serve as a modulator of abdominal adipogenesis by sponging gga-miR-301b-3p in chickens.miRNAs function as regulatory molecules that recognize 3′ UTRs of protein-coding genes to post-transcriptionally trigger their cleavage or translational repression [59]. Therefore, we predicted major targets of gga-miR-301b-3p and confirmed *ACSL1* gene as a direct target of gga-miR-301b-3p. Meanwhile, *ACSL1* mRNA abundance was suppressed by gga-miR-301b-3p overexpression and enhanced by gga-miR-301b-3p knockdown. Strikingly, the inhibitory effects of gga-miR-301b-3p on the post-transcriptional expression of *ACSL1* were relieved by circDOCK7 in both proliferative and differentiated chicken abdominal preadipocytes, and the above-mentioned promoted role of circDOCK7 in *ACSL1* mRNA expression was abolished by gga-miR-301b-3p, indicating that circDOCK7 could serve as a ceRNA to sponge gga-miR-301b-3p and prevent *ACSL1* gene from gga-miR-301b-3p-mediated mRNA degradation, thereby post-transcriptionally upregulating *ACSL1* expression. Notably, the *ACSL1* gene belongs to the *ACSL* gene family that is responsible for catalyzing the conversion of exogenous or endogenous long-chain fatty acids by adipose cells into acyl coenzyme A (acyl-CoA), which undergoes β-oxidation in the mitochondrial matrix for the formation of acetyl-CoA, a raw lipid synthesis precursor that can then be incorporated into triglycerides, phospholipids, and cholesteryl esters, and has been proven to be at the critical juncture of de novo lipid synthesis and β-oxidation degradation [60]. ACSL1 has been identified as a dominant ACSL isoform, owing to its 80% contribution to the total ACSL activity in the adipose tissue, and preferentially utilizes palmitoleate, oleate, and linoleate as substrates [61, 62]. The *ACSL1* gene showed the highest mRNA abundance in adipose tissue of mammals and Chinese indigenous Lushi blue-shell-egg chickens [63], which is in agreement with the finding of our present study that *ACSL1* is most abundantly expressed in the adipose tissue of commercial broilers. In addition, in vivo and in vitro expression patterns showed a noteworthy increase in *ACSL1* gene expression in the abdominal fat of chickens with high abdominal fat percentage and during the proliferation and adipogenic differentiation of chicken abdominal preadipocytes in a time-dependent manner. Moreover, ACSL1 has been reported to be localized in the mitochondrial membrane, plasma membrane, and endoplasmic reticulum of mammalian adipocytes [64]. The endoplasmic reticulum is a major “lipid factory” organelle where lipids are synthesized, assembled, and transported. Mitochondria are crucial sites for free fatty acid β-oxidation and glycolysis and can import certain proteins and lipids to maintain cell survival [65]. Our subcellular localization analysis also showed that chicken ACSL1 was localized in the endoplasmic reticulum and mitochondria in chicken abdominal preadipocytes. Functionally, the adipose-specific *ACSL1* knockout mice exhibited a 50%–90% lower fatty acid oxidation rate in adipocytes isolated than that in control adipocytes [61]. In adipocytes differentiated from mesenchymal stem cells of neonates born small for gestational age, both the mRNA and protein abundance of the *ACSL1* gene consistently increased during adipogenesis, in parallel with enhanced glucose uptake and total lipid content, and *ACSL1* deficiency impeded lipid loading and reduced glucose uptake [66]. Despite this evidence concerning the importance of *ACSL1* in mammalian adipogenesis, little is known about the role of *ACSL1* in avian adipogenesis. Consequently, we first detected the effects of *ACSL1* gene on the proliferation and adipogenic differentiation of chicken abdominal preadipocytes using gain- and loss-of-function assays and found that the overexpression of *ACSL1* gene contributed to a significant increase in proliferation concomitant with their maturation into adipocytes, characterized by increased intracellular lipid droplet accumulation and triglyceride and acetyl-CoA levels, and vice versa. During cell proliferation, proliferating cells often display enhanced uptake of glucose, an energy source for ATP generation, as well as an important carbon source to support the production of lipids and biosynthesis of nucleotides and non-essential amino acids to produce daughter cells. Heavy consumption of glucose is essential for nucleotide biosynthesis and successful replicative cell division for cancer cell growth [67]. It has been reported that low glucose could induce cell cycle G1 arrest to inhibit the proliferation of human endometrial cancer cell lines [68]. Likewise, our results showed that ACSL1 improved the glucose uptake capacity of proliferating chicken abdominal preadipocytes. Therefore, we concluded that the *ACSL1* gene could serve as a fundamental contributing factor in abdominal fat deposition by facilitating the proliferation, at least by promoting glucose uptake capacity, and adipogenic differentiation of abdominal preadipocytes in chickens.

New insights have been gained regarding the direct RNA-RNA interactome mediated by circRNAs to regulate the biogenesis and function of RNA molecules other than miRNAs, such as long non-coding RNAs and mRNAs, to influence their stability, translation, and localization [69]. A recent study concerning the interaction of exonic circRNA with mRNA reported that loop-hairpins were required for the circRNA-mRNA interactome, and an immune cell-associated circRNA circPan3 could promote the mRNA expression of the interleukin 13 receptor subunit alpha 1 (*IL-13Rα1*) gene upon facilitation of its mRNA stability through direct interaction with *IL-13Rα1* mRNA [70]. RNA secondary structure prediction based on the circDOCK7 sequence showed that circDOCK7 also has a loop-hairpin structure. Therefore, we speculated that circDOCK7 might directly interact with key genes involved in lipid metabolism to modulate their stability, localization, and/or expression, and consequently regulate abdominal fat deposition in chickens, which needs to be further investigated.

## Conclusions

**Fig. 12:**
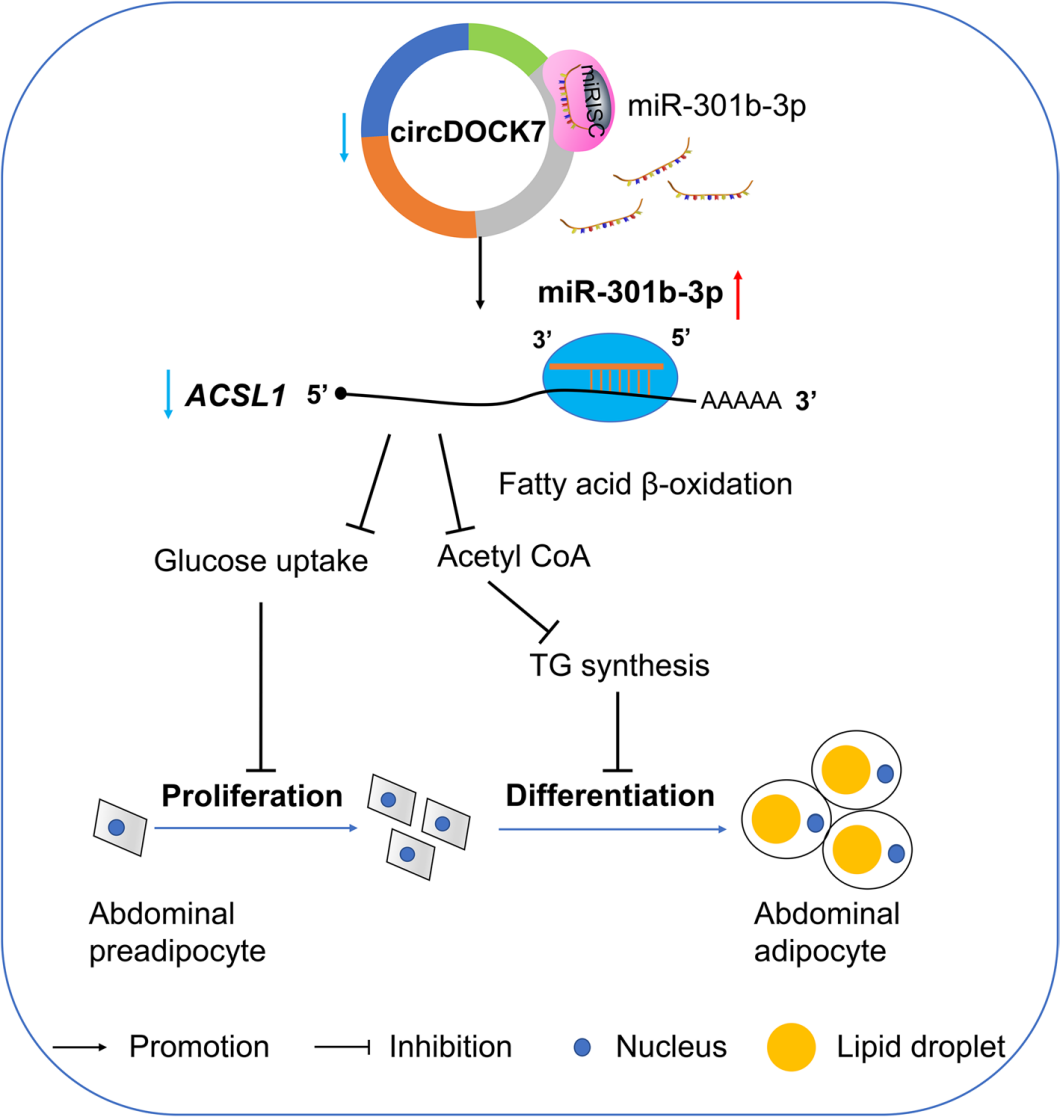
Proposed model for the regulatory mechanism of circDOCK7 in chicken abdominal adipogenesis. CircDOCK7 is a new adipogenesis-stimulating circRNA in chickens. CircDOCK7 serves as a ceRNA to modulate the gga-miR-301b-3p/*ACSL1* axis, thus promoting the proliferation and adipogenic differentiation of chicken abdominal preadipocytes

In conclusion, a cytoplasmic exonic circDOCK7 could function as a miRNA sponge that directly sequesters gga-miR-301b-3p away from the *ACSL1* gene to post-transcriptionally promote *ACSL1* mRNA expression, subsequently facilitating the proliferation and differentiation of abdominal preadipocytes in chickens (Fig. 12). To the best of our knowledge, this is the first study to provide strong evidence that circRNAs involved in abdominal fat deposition act as miRNA sponges in chickens, shedding new light on regulatory mechanisms in avian adipogenesis.

## Supplementary Information

**Additional file 1:** **Table S1.** Primer information used in this study. **Table S2.** Sequences of circDOCK7 transcripts.

**Additional file 2:** **Fig. S1.** Predicted secondary structure of circDOCK7. **Fig. S2.** Effects of circDOCK7 overexpression on the proliferation of chicken abdominal preadipocytes. **Fig. S3.** Prediction of m^6^A modification sites based on circDOCK7 sequence. **Fig. S4.** Effects of circDOCK7 on the expression of its parental *DOCK7* gene. **Fig. S5.** CircDOCK7-mediated potential ceRNA regulation during the adipogenic differentiation of chicken abdominal preadipocytes. **Fig. S6.** Prediction of subcellular localization of chicken ACSL1 protein using UniProt.

## Data Availability

The data for the current study are available from the corresponding author upon reasonable request.

## Abbreviations

Acetyl-CoA: Acetyl coenzyme A
ACSL1: Acyl-CoA synthetase long-chain family member 1
CCK8: Cell Counting Kit-8
cDNA: Complementary DNA
CEBPα: CCAAT/enhancer-binding protein alpha
ceRNA: Competing endogenous RNA
CircRNA: Circular RNA
DMEM-F12: Dulbecco’s modified Eagle’s medium F12
DOCK7: Dedicator of cytokinesis 7
EdU: 5-Ethynyl-2-deoxyuridine
ER: Endoplasmic reticulum
FBS: Fetal bovine serum
gDNA: Genomic DNA
HAbF: High abdominal fat percentage
ICP2: Immortalized chicken preadipocytes 2
IRES: Internal ribosome entry site
LAbF: Low abdominal fat percentage
miRNAs: MicroRNAs
MT: Mitochondria
ORFs: Open reading fragments
PPARγ: Peroxisome proliferator activated receptor gamma
qRT-PCR: Quantitative real-time PCR
TG: Triglycerides
3' UTR: 3' Untranslated region
