# Supplementary material for: CircDOCK7 facilitates the proliferation and adipogenic differentiation of chicken abdominal preadipocytes through the gga-miR-301b-3p/ACSL1 axis
Source: J Anim Sci Biotechnol. 2023 Jul 6;14:91. doi: 10.1186/s40104-023-00891-8 (PMC10324207; doi:10.1186/s40104-023-00891-8)
Supplement: Supplementary file 1 — Additional file 1: Table S1. Primer information used in this study. Table S2. Sequences of circDOCK7 transcripts. [file 40104_2023_891_MOESM1_ESM.docx]

**Table S1** Primer information used in this study

| **Primers’ names** | **Primer sequences (5’→3’)** | **Product length** | **Usage** |
| --- | --- | --- | --- |
| circDOCK7-F | CAAGCCTTAACCTGAACCGC | 186 bp | Divergent primers  qRT-PCR analysis |
| circDOCK7-R | GGAGGGACGATACAGCAACAA |  |  |
| *DOCK7*-convergent primer-F | TTTGCACTTGTCCATGCTCTG | 203 bp | Convergent primer |
| *DOCK7*-convergent primer-R | CCTGCAATGACAGGAGGTCTT |  |  |
| *DOCK7*-F | TTGGCACCTACTTCAGGGTC | 239 bp | qRT-PCR analysis |
| *DOCK7*-R | AGGCTCCACGTAGGTGATCT |  |  |
| *PPARγ*-F | GGAGATCGCCCAGGTTTGTT | 184 bp |  |
| *PPARγ*-R | CTGCACGTGTTCCGTTACAA |  |  |
| *CEBPα*-F | AGAACGAGCACTCCATCGAC | 134 bp |  |
| *CEBPα*-R | GAAATCGAAATCCCCGGCCA |  |  |
| *ACSL1*-F | TACCCTGGTGGGTTTTGGTG | 144 bp |  |
| *ACSL1*-R | AGGAGAGAGGACCTTCGAGC |  |  |
| *GAPDH*-F | AGAACATCATCCCAGCGT | 182 bp |  |
| *GAPDH*-R | AGCCTTCACTACCCTCTTG |  |  |
| gga-miR-301b-3p-F | GCAGTGCAATAGTATTGTCAAAG |  | qRT-PCR analysis |
| U6-F | CACGCAAATTCGTGAAGCGTTCCA |  |  |
| circDOCK7-WT-F | GCAGTAATTCTAGGCGATCGCTCGAGAGTTCGCTCCATCATTGGCA |  | Dual luciferase reporter plasmids construction |
| circDOCK7-WT-R | AAGATATTTTATTGCGGCCAGCGGCCGCAGAAATCGCACTACCGGCTC |  |  |
| circDOCK7-Mut-F | CCCTTATCTTGACAAGTTCTTGTCCATGCTCTGGATGAGC |  |  |
| circDOCK7-Mut-R | GCTCATCCAGAGCATGGACAAGAACTTGTCAAGATAAGGG |  |  |
| ACSL1-3’UTR-WT-F | GCAGTAATTCTAGGCGATCGCTCGAGTGGCACTGGATGGGTATTTGT |  |  |
| ACSL1-3’UTR-WT-R | AAGATATTTTATTGCGGCCAGCGGCCGCGAAATAGCCAACACTAAGCAAACA |  |  |
| ACSL1-3’UTR-Mut-F | GATACAAGCACAACAAGGTAAGATTTGTGTGATTGTAA |  |  |
| ACSL1-3’UTR-Mut-R | TTACAATCACACAAATCTTACCTTGTTGTGCTTGTATC |  |  |
| pcDNA3.1-ACSL1-F | CTATAGGGAGACCCAAGCTGGCTAGCATGATGCAAGCACACGACTTG |  | Overexpression vector construction |
| pcDNA3.1-ACSL1-R | CCGCGGTACCGTCGACTGCAGAATTCCCATTTTGGTATTAGCATAGAG |  |  |
| pLC5-ciR-circDOCK7-F | cgGAATTCTAATACTTTCAGGTTCCCCTTCCTGGAATGAA |  |  |
| pLC5-ciR-circDOCK7-R | cgGGATCCAGTTGTTCTTACCTTACTGCCAATGATGGAGC |  |  |

*F* refers to forward primer; *R* refers to reverse primer

The letters marked in red mean the homologous sequences to corresponding vector; the underlined letters mean restriction enzyme sequences; lowercase letters mean the protective bases for restriction enzyme; the letters marked in blue mean the forward cyclization mediated sequences; the letters marked in orange mean the reverse cyclization mediated sequences; the AG letters marked in purple mean AG acceptor, and the AC letters marked in purple mean GT donor

**Table S2** Sequences of circDOCK7 transcripts

>circDOCK7

GTTCCCCTTCCTGGAATGAAGTGGGTGGATAATCACAAAGGAGTTTTCAATGTAGAAGTTGTTGCTGTATCGTCCCTCCATACTCAGGACCCTTATCTTGACAAGTTCTTTGCACTTGTCCATGCTCTGGATGAGCACATGTTCCCTGTGCGAATAGGAGACATGAGAATTATGGAAAACAACTTGGAAAATGAACTGAAGAGCAGCATTTCAGCTTTAAATTCCTCTCAACTGGAGCCGGTAGTGCGATTTCTTCATCTCCTGCTTGACAAACTGATTCTTCTGGTAGTAAGACCTCCTGTCATTGCAGGCCAAATAGTTAACCTTGGCCAAGCATCTTTTGAAGCAATGGCTTCAATCATAAACAGACTTCACAAGAACCTGGATGGAAACCAGGACCAGCATGGCAGAAACAGCCTTCTTGCTTCCTATATTTATTATGTTTTTCGCCTACCAAATACCTATCCCAACTCACCATCACCAGGTCCCGGAGGCTTAGGGGGATCAGTGCATTATGCCACCATGGCTCGATCCGCTGTCCGACCTGCAAGCCTTAACCTGAACCGCTCCCGCAGCCTCAGCAACAGCAACCCAGATATATCTGGGACCCCCACCTCACCTGACGATGAAGTTCGCTCCATCATTGGCAGTAAG

Note: the back-splicing junctions were highlighted in red
