# Supplementary material for: CircDOCK7 facilitates the proliferation and adipogenic differentiation of chicken abdominal preadipocytes through the gga-miR-301b-3p/ACSL1 axis
Source: J Anim Sci Biotechnol. 2023 Jul 6;14:91. doi: 10.1186/s40104-023-00891-8 (PMC10324207; doi:10.1186/s40104-023-00891-8)
Supplement: Supplementary file 2 — Additional file 2: Fig. S1. Predicted secondary structure of circDOCK7. Fig. S2. Effects of circDOCK7 overexpression on the proliferation of chicken abdominal preadipocytes. Fig. S3. Prediction of m6A modification sites based on circDOCK7 sequence. Fig. S4. Effects of circDOCK7 on the expression of its parental DOCK7 gene. Fig. S5. CircDOCK7-mediated potential ceRNA regulation during the adipogenic differentiation of chicken abdominal preadipocytes. Fig. S6. Prediction of subcellular localization of chicken ACSL1 protein using UniProt. [file 40104_2023_891_MOESM2_ESM.docx]

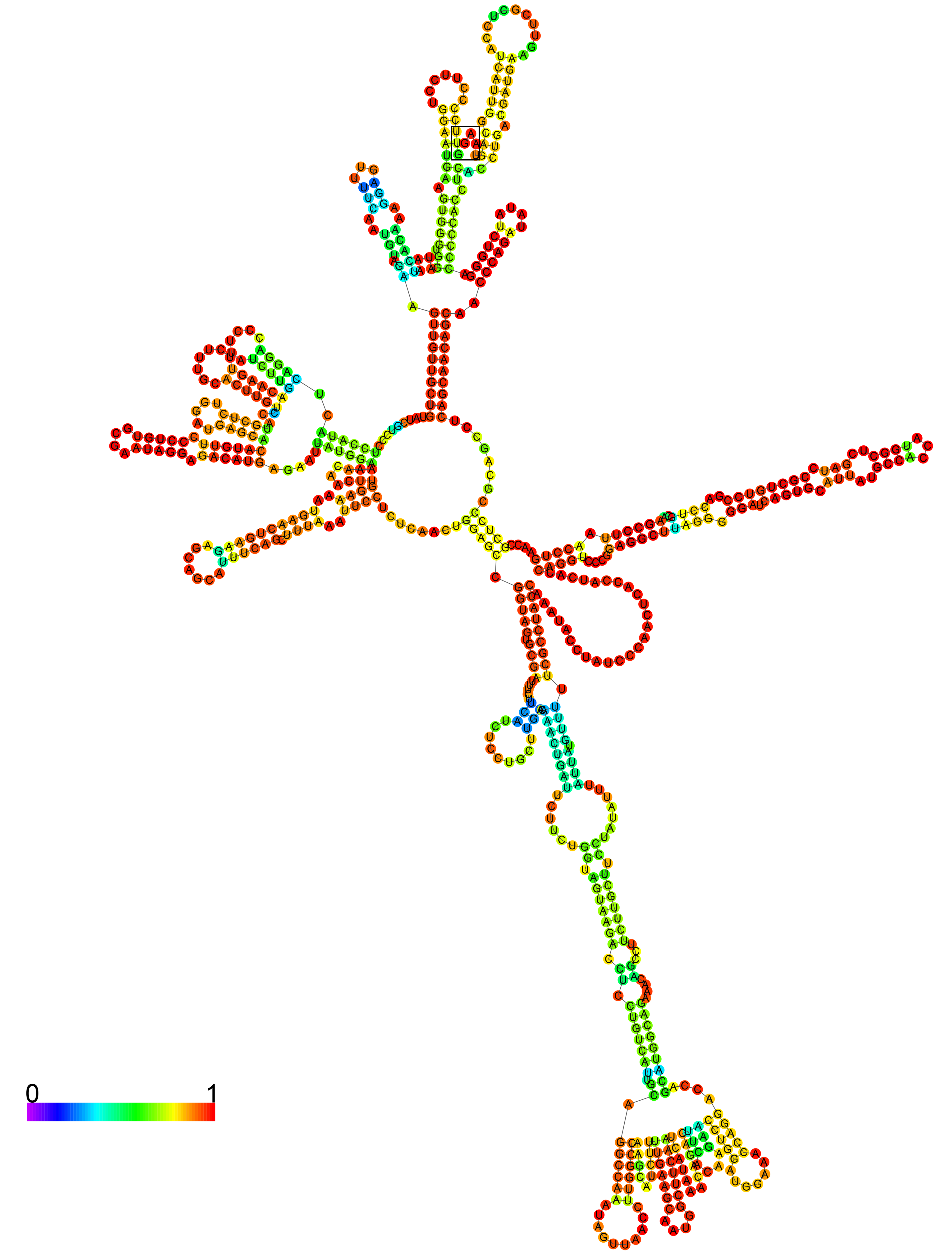


**Fig. S1** Predicted secondary structure of circDOCK7. The black rectangle indicates the backsplicing junction sites


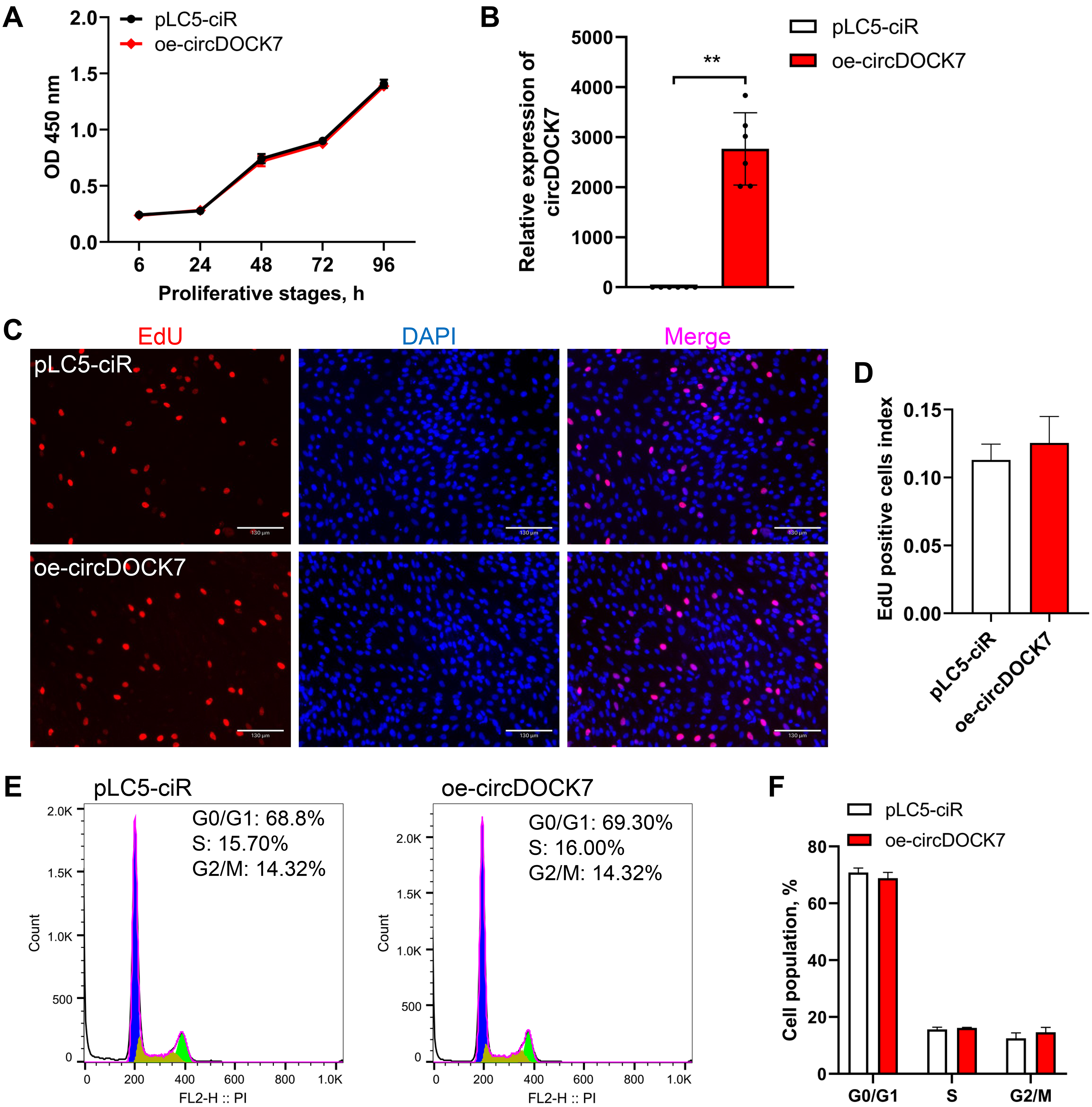


**Fig. S2** Effects of circDOCK7 overexpression on the proliferation of chicken abdominal preadipocytes. **A** CCK8 assay of chicken abdominal preadipocytes transfected with the pLC5-ciR empty vector and circDOCK7 overexpression vector at 6, 24, 48, 72, and 96 h post-transfection; **B** Detection of circDOCK7 overexpression in chicken abdominal preadipocytes after 48 h of transfection with the circDOCK7 overexpression vector; **C** Proliferation of chicken abdominal preadipocytes determined by the EdU staining assay after 48 h of transfection with the circDOCK7 overexpression vector; **D** Histogram showing the proportion of EdU-positive cells using ImageJ; **E** and **F** Flow-cytometric cell cycle analysis of chicken abdominal preadipocytes after 48 h of transfection with the circDOCK7 overexpression vector


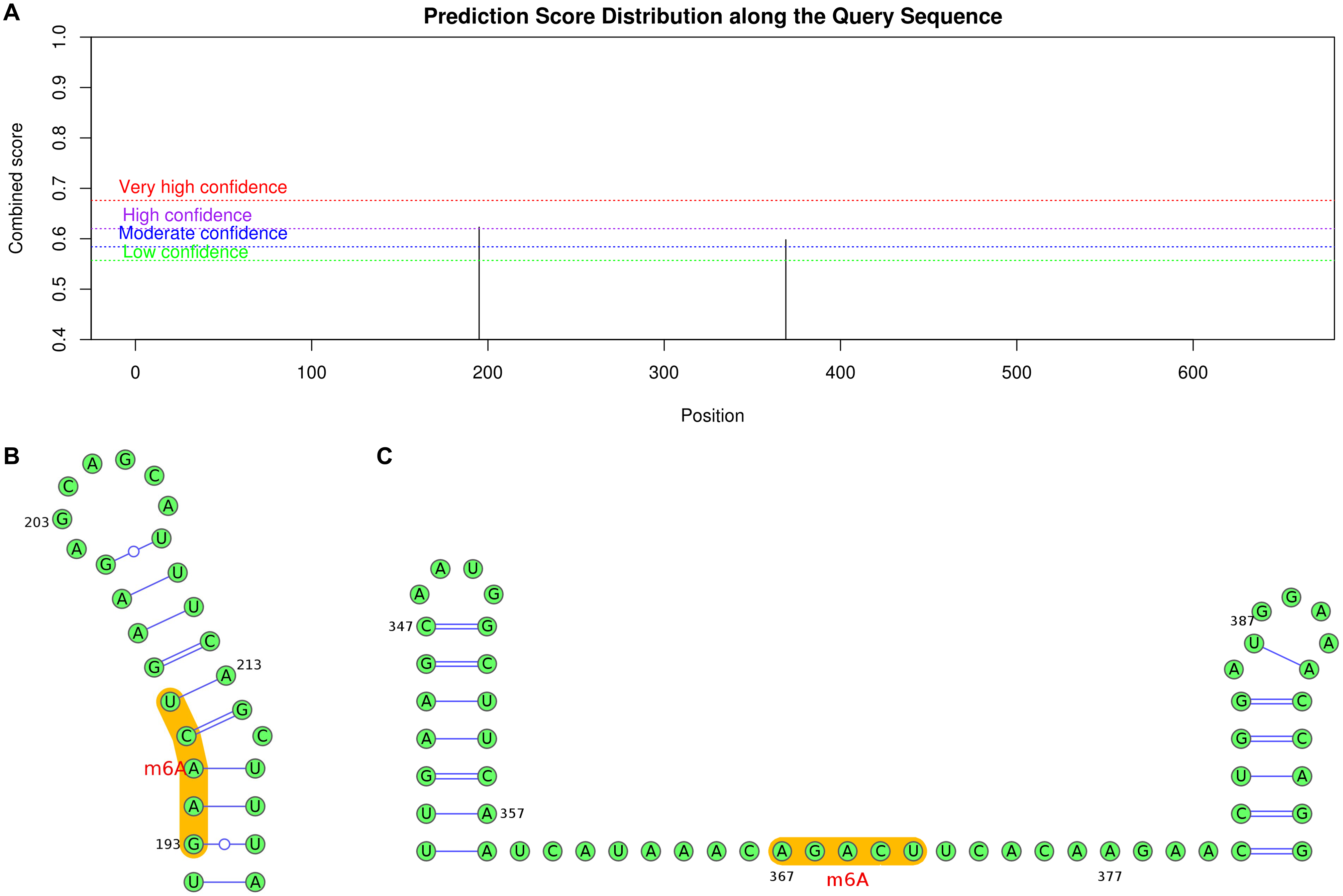


**Fig. S3** Prediction of m^6^A modification sites based on circDOCK7 sequence. **A** Overview of the predicted m^6^A modification sites of circDOCK7; **B** and **C** Graphical representation of the local secondary structure context around positions 195 and 369 of m^6^A modification sites


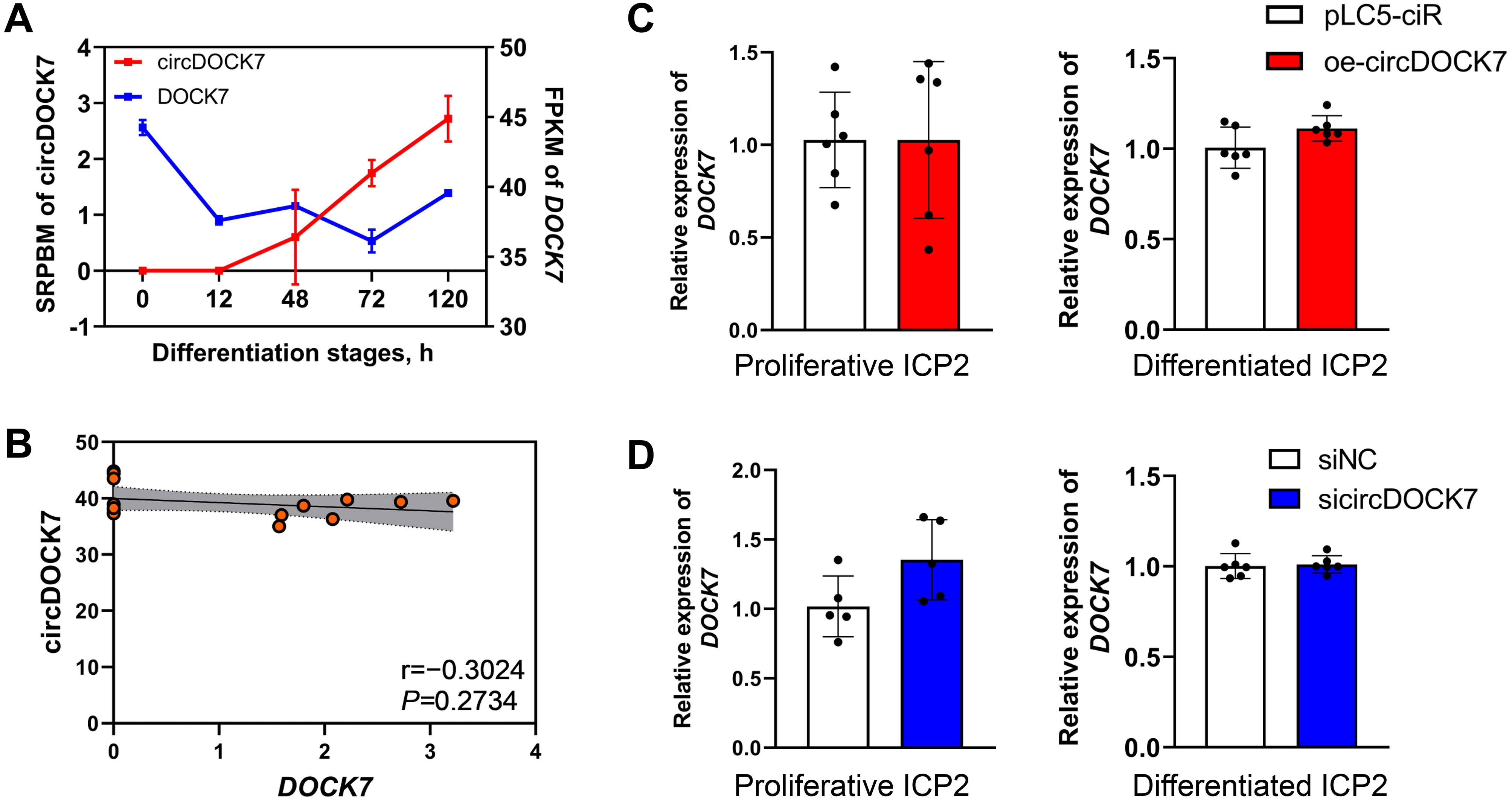


**Fig. S4** Effects of circDOCK7 on the expression of its parental *DOCK7* gene. **A** RNA sequencing data-based expression pattern analysis of circDOCK7 and *DOCK7* gene in chicken abdominal preadipocytes at different adipogenic differentiation stages; **B** RNA sequencing data-based correlation analysis of the expression levels of circDOCK7 and *DOCK7* gene during the adipogenic differentiation of chicken abdominal preadipocytes; **C** and **D** Relative expression of the *DOCK7* gene in proliferative and differentiated chicken abdominal preadipocytes upon circDOCK7 overexpression and knockdown


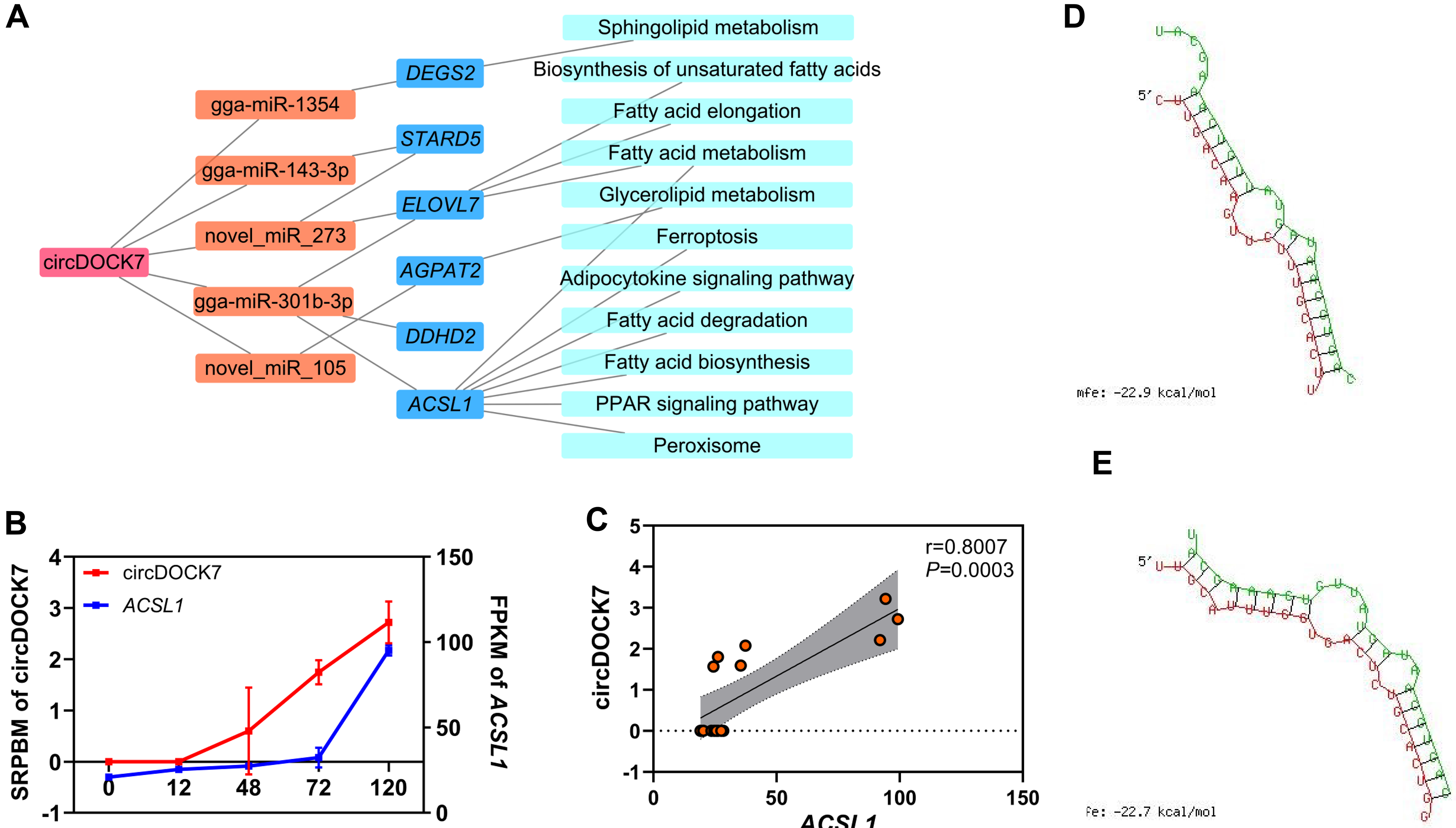


**Fig. S5** CircDOCK7-mediated potential ceRNA regulation during the adipogenic differentiation of chicken abdominal preadipocytes. **A** circRNA-miRNA-mRNA ceRNA network consisting of circDOCK7, differentially expressed miRNA, and lipid-related genes; **B** RNA sequencing data-based expression pattern analysis of circDOCK7 and *ACSL1* gene in chicken abdominal preadipocytes at different adipogenic differentiation stages; **C** RNA sequencing data-based correlation analysis of the expression levels of circDOCK7 and *ACSL1* gene during the adipogenic differentiation of chicken abdominal preadipocytes; **D** Secondary structure of the RNA duplex of circDOCK7 and gga-miR-301b-3p. Red indicates circDOCK7; green indicates gga-miR-301b-3p. **E** Secondary structure of the RNA duplex of gga-miR-301b-3p and the 3′ UTR of the *ACSL1* gene. Red indicates the *ACSL1* gene; green indicates gga-miR-301b-3p

**
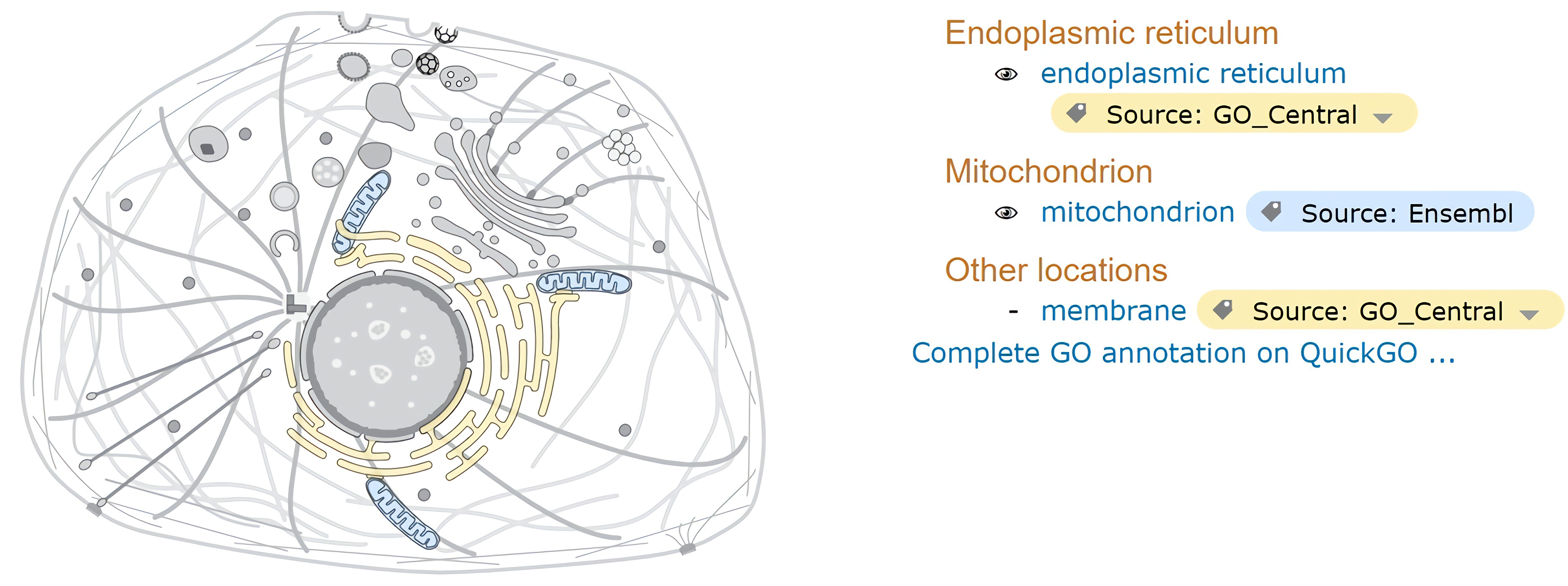
**

**Fig. S6** Prediction of subcellular localization of chicken ACSL1 protein using UniProt
